# Improving Trial Informativeness: A Rapid Review of Global Research on How to Ensure Trials Are Useful

**DOI:** 10.1111/jep.70147

**Published:** 2025-06-11

**Authors:** Sarah R. Prowse, Shaun Treweek, Miriam Brazzelli, Hanne Bruhn

**Affiliations:** ^1^ Aberdeen Centre for Evaluation University of Aberdeen Aberdeen UK

## Abstract

**Rationale:**

Randomised controlled trials are considered the ‘gold standard’ in clinical research and decision‐making. However, many trials have significant flaws that current review processes fail to identify early enough for corrections to be made. Flaws in trial design, conduct and reporting ultimately lead to research waste. This rapid review provides insights from global research aimed at improving trial ‘informativeness’ as described by Zarin and colleagues.

**Methods:**

A rapid review was conducted with a focus on research addressing trial design processes that might improve informativeness aligned with one or more of the five key conditions outlined by Zarin and colleagues: 1) Importance, 2) Design, 3) Feasibility, 4) Integrity and 5) Reporting. A further thematic analysis was conducted using NVivo 12.

**Results:**

The final review includes 42 texts. Of the 27 recommended processes or actions to improve trial informativeness, most were relevant to the second condition of trial design (2) Design; 44%). A key recommendation was the use of ‘tools’ to enhance trial informativeness. A total of 23 tools were identified across the conditions of 1) Importance (17%), 2) Design (74%) and 5) Reporting (9%).

**Conclusion:**

This review highlights how a better understanding of design processes that lead to informative trials can reduce or eliminate research waste. Further research is needed on how these processes can better support pre‐funding peer review, which would also increase the likelihood of producing informative trials.

## 1 Introduction

Randomised controlled trials (RCTs) are considered the ‘gold standard’ in research and decision‐making, particularly in healthcare, social science and policy interventions. However, many trials have significant flaws that current review processes fail to identify early enough for corrections to be made. Flaws in trial design, conduct and reporting lead to research waste by failing to advance scientific knowledge and by failing to provide a demonstrable return on the resources invested throughout a trial process [1, 2, 3].

The COVID‐19 pandemic brought attention to the issue of research waste and trials that do not provide useful information for clinical decision‐making [4]. A 2020 study found that 42% of COVID‐19 trials conducted in western Europe were poorly designed and contributed to research waste [5]. Moreover, poorly planned and executed trials raise ethical concerns as participants may be exposed to unnecessary risk without any potential benefit [6, 7].

In 2019, Zarin and colleagues introduced the concept of ‘uninformative trials’ to describe trials that are designed, conducted and reported in ways that fail to fulfil participants' expectations that their involvement will advance medical science [8]. In contrast, Zarin and colleagues further described ‘informative trials’ as meeting with five key conditions for *informativeness* [8, 9]:

1. Importance: the trial hypothesis is likely to inform an important scientific, medical, or policy decision;
2. Design: the trial methods are likely to provide meaningful evidence related to the study hypothesis;
3. Feasibility: the trial must be demonstrably feasible (e.g., it must have a realistic plan for recruiting sufficient participants);
4. Integrity: the trial must be conducted and analysed in a scientifically valid manner that is faithful to the design; and
5. Reporting: systems are in place to ensure timely, complete and accurate reporting.

A further study on the conditions for informativeness by Hutchinson and colleagues concluded that most trials designed to guide clinical practice had features that could undermine their ability to do so [9]. While some trial funders or sponsors have post‐funding scientific review processes, pre‐funding peer review remains the primary method for evaluating non‐industry trials. Therefore, increasing the number of informative trials also requires improving pre‐funding review processes.

This rapid review provides insights from global research aimed at improving informativeness in line with the five conditions described by Zarin and colleagues, particularly where that work presents recommendations for improvement. Understanding these processes could enhance pre‐funding peer review, increasing the likelihood of producing informative trials.

## 2 Methods

We conducted a rapid review informed by methodological guidance from the Cochrane Rapid Reviews Methods Group with an emphasis on maintaining systematic, transparent and reproducible methods through an accelerated review process [10]. A protocol for the review was developed and published on PROSPERO (CRD42024541229) [11].

### 2.1 Eligibility Criteria

Research for inclusion addressed trial design processes that might be expected to improve informativeness, such as one or more of Zarin and colleagues' five conditions: 1) Importance, 2) Design, 3) Feasibility, 4) Integrity, and 5) Reporting [8]. This included any proposed or evaluated approach to improve trial design that might increase the chance of an informative trial, using any methodology. The full inclusion and exclusion criteria for the review are available via the protocol [11].

### 2.2 Information Sources and Search Strategy

A comprehensive search strategy was developed and piloted with the assistance of an information specialist and is presented in Supporting Information S1: Supporting Material 1. MEDLINE (Ovid) was searched for relevant literature from January 2014 to May 2024 when the search was conducted. No language restrictions were initially applied with English language used as a final limiting filter. Due to the timeframe of this rapid review, searches were not re‐run prior to the final analysis and unpublished studies were not sought.

### 2.3 Study Selection

All references identified by the search strategy were uploaded to both EndNote X9 and the Evidence for Policy and Practice Information software programme (EPPI‐Reviewer 5) for further removal of duplicates. EPPI‐Reviewer is recommended by Cochrane for all forms of reviewing and utilises machine learning to rapidly advance the screening process [12].

After the removal of duplicates, manual sifting of titles and abstracts was performed by one reviewer (S. P.) on 20% of retrieved citations. This allowed for the use of a classifier model within the EPPI‐Reviewer software to identify further citations that the software judged to have an inclusion probability of 50% or greater based on the results of the 20% sample.

A 10% manual screening was undertaken by two reviewers (S. P. and S. T.) of articles placed by EPPI‐Reviewer in the lower probability bands of 40%–50% and 30%–40% where agreement was reached that no relevant citations had been excluded and that EPPI‐Reviewer was identifying citations appropriately. Citations for full‐text retrieval were agreed upon by three reviewers (S. P., S. T., and H. B.). Full texts were then assessed by one reviewer (S. P.), with a second reviewer (S. T.) assessing 10% to reach consensus on inclusion within the review.

### 2.4 Data Extraction

A data extraction form was developed to capture research specifics, including citation details, geographic scope, funding details, the type of intervention or proposed intervention, relevance to Zarin and colleagues, and key outcomes or anticipated outcomes. The data extraction forms are presented in Supporting Information S2: Supporting Material 2.

### 2.5 Synthesis and Analysis

We decided a priori to acknowledge the heterogeneous nature of the research, and to be guided by the evidence presented by the authors of the included literature when considering the diverse processes influencing the informativeness of trials. The full texts of citations identified for inclusion were uploaded to NVivo 12 for further thematic analysis.

Zarin and colleagues' five conditions for informativeness (1) Importance, 2) Design, 3) Feasibility, 4) Integrity and 5) Reporting) were used for thematic analysis. A reviewer (S. P.) identified and coded examples of relevant processes (proposed or implemented) from the literature according to each theme and key findings were summarised in tabular form. Additional supporting exemplars of these processes, or suggested tools to support informative trial design, were also collected for further synthesis and discussion. As this is a rapid review covering research in varying contexts, no quantitative or meta‐analytical synthesis of findings was undertaken.

## 3 Results

**Figure 1:**
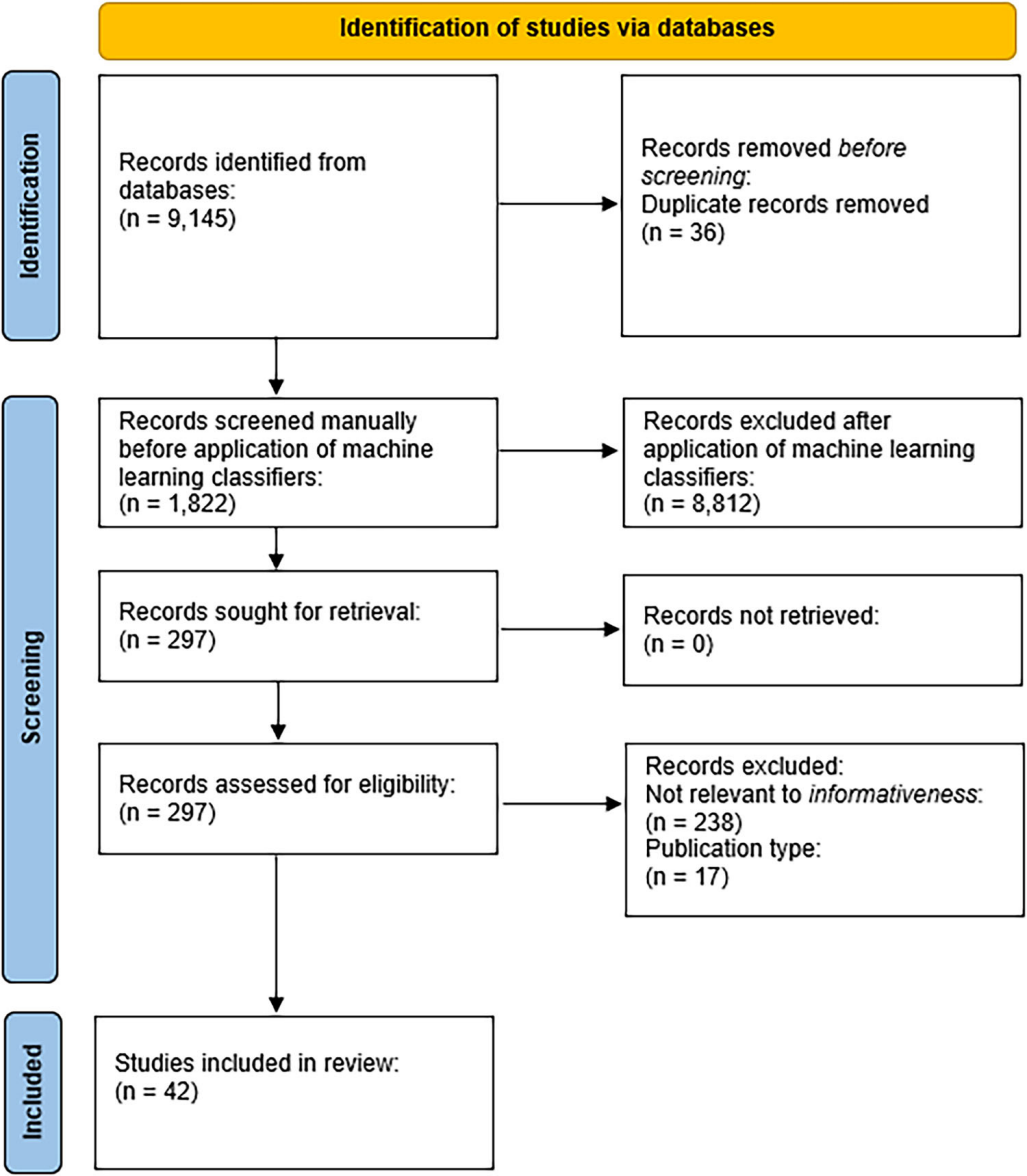
Results of the publication selection process in a PRISMA‐style diagram.

Figure 1 summarises the results of the publication selection process in a PRISMA‐style diagram. A total of 42 full‐text articles were included in the final review.

### 3.1 Overview of the Included Texts

Table 1 summarises the geographic setting of the research, or the geographic affiliations of publishing authors, and predominantly reflects research from the United States of America (USA; 28.6%) and the United Kingdom (UK; 21.4%). Research with a global focus (11.9%) covered three or more distinct geographic areas.

**Table 1:** Overview of included texts by geography and funder.

Geographic setting of the research (or, geographic affiliations of publishing authors)	% (*n* = 42)^a^
United States of America	28.6%
United Kingdom	21.4%
Global (studies comprised of three or more distinct geographic areas)	11.9%
Sub‐Saharan Africa	7.1%
Europe (broadly)	7.1%
Europe + United Kingdom	7.1%
Canada + United Kingdom	7.1%
Europe + United States of America	4.8%
Australia + New Zealand	2.4%
Canada + United States of America	2.4%

Funder and/or sponsor of the research	% (*n* = 42)^a^
Government	31%
Funding not stated	19%
No specific funding received	16.7%
Charity	9.5%
Charity + government	7.1%
Industry	4.8%
Research council	2.4%
Charity + academic	2.4%
Academic + government + research council	2.4%
Government + other collaborative network	2.4%
Other collaborative networks	2.4%

^a^ May not equal 100% due to rounding.

Table 1 also details the funder and/or sponsor of the research. Approximately one‐third of the research was sponsored by global government agencies (31%) with the United States Food and Drug Administration being the largest individual named sponsor (4.8%). Funding information was not provided for 19% of the research captured, while 16.7% of publications provided further transparency by stating that no specific funding had been received.

### 3.2 Improving Informativeness Through Trial Design Processes

A total of 27 recommended processes or actions to improve informativeness were captured by the rapid review. Most of these recommendations were relevant to Zarin and colleagues' second condition for informativeness—2) Design (~44%). Table 2 summarises the recommendations alongside further supporting descriptors and examples from the body of research.

**Table 2:** Summary of recommended processes or actions to improve trial informativeness.

**Recommended process or action to improve informativeness**	**Supporting examples from the literature**
**1.** ***Importance: trial hypothesis is likely to inform an important scientific, medical or policy decision***	
1.1. Implement a formal process of research priority or agenda setting that leverages existing structures and systems	Identification and prioritisation of research questions through evidence synthesis (including systematic reviews, scoping reviews and other forms of evidence mapping [13, 14, 15, 16]), Value of Information (VOI) and other economic analyses [17, 18], and stakeholder consultations [19]
1.2. Develop fit‐for‐purpose (e.g., aligned with study intent) quality enhancement initiatives to support the early conceptualisation of research questions	Consider if current quality definitions and frameworks (e.g., INQUIRE [20]) within trials research are aligned with the study intent and how quality enhancement initiatives may differ for low‐resource settings [21, 22]
1.3. Include patients as partners at the outset of the trial development process	Emphasis on the role of patients in guiding the development of trial hypotheses to better address issues important to patients and so that the proposed intervention is more likely to be seen by patients as acceptable [23, 24]
1.4. Extend and formalise traditional research practices through Open Science (OS)	Pre‐registering prospective projects on online platforms or submitting a registered report where hypotheses, experimental design and analytic plans are specified before data collection [25, 26]
**2.** ***Design: trial methods are likely to provide meaningful evidence related to the study hypothesis***	
2.1. Select fit‐for‐purpose tools to further support a process of informed trial design	Trial designs can be further improved through the use of appropriate design tools (see Table 3) as well as end‐to‐end management platforms (e.g., from trial inception through final reporting) [22] and frameworks or other guiding documents intended to reduce research waste [19, 27, 28, 29]
2.2. Establish research forums and other collaborative networks as a source of trial design feedback and support	Research forums and other collaborative networks can provide useful feedback when considering trial design and conduct, such as obtaining approvals, data management and developing good work relationships with funders [30, 31, 32]; low‐resource settings may also uniquely benefit from further collaboration when considering trial design and implementation [33]
2.3. If appropriate, consider pragmatic trial design approaches that evaluate the effectiveness of interventions under real‐world conditions	Design tools such as PRECIS‐2 [34] and the GetReal Trial Tool [35] can help conceptualise and inform both explanatory and/or pragmatic elements of trial design with the intent of creating trials that match design decisions to the intended decision‐making purpose of the trial
2.4. Integrate a patient‐centric approach throughout the trial design process (e.g., weighing participant considerations such as convenience, risk to benefit ratio, social interaction, partnership and altruism)	Consideration of patient needs, notably in early phase trial development, can lead to fewer protocol amendments, improved endpoints, improved feasibility (recruitment and retention) and higher patient satisfaction [24, 36]
2.5. Consider a diversity of expertise within trial teams to ensure conditions for informativeness are incorporated throughout all aspects of trial planning and execution	Expertise should be sought across all areas of trial design and development including information technology, data development, scientific protocol development, clinical affairs and financial operations; diversity is notably relevant to multicentre trials where expertise may span multiple teams working to achieve a shared outcome [36, 37]
2.6. Seek further protocol guidance and feedback from anticipated trial site staff and team members	Site staff involvement in protocol development can further understanding of local context, capacity and culture as well as ensuring that anticipated team members understand the protocol before trial initiation [36]
2.7. Use existing registry data to further inform clinical trial design	Potential applications of existing registry data include informing aspects of trial design such as sample size [38], or the use of additional tools to predict the early termination of a trial based on previous outcomes [39, 40]
2.8. Further deliberation of critical ethical issues within trial protocol development	Substantive discussion of specific ethical issues is rarely included in clinical trial protocols and current reporting guidelines (e.g., SPIRIT [41], CONSORT [42]) may not adequately support protocol writers, study teams, sponsors, ethics committees and reviewers in adequately addressing ethical issues [43]
2.9. Accountability from trial funders and/or sponsors to ensure trial design considerations are appropriate and adequately justified	Trial funders and/or sponsors should question the selection and validation of outcomes, interventions and comparators, sample sizes and suggested follow‐up within a trial design; validation scales (e.g., COMET [44]) may assist in choosing appropriate measures [45]
2.10. Utilise benefit‐risk assessments for trials with more than one outcome of interest (e.g., superiority, equivalence and non‐inferiority trials)	Benefit‐risk methodologies could be used to assess outcomes simultaneously and consider trade‐offs while helping to ensure research hypotheses are answered appropriately (e.g., the effectiveness of the primary health outcome, relative to safety and cost) [46]
2.11. Conduct a scientific design review after the peer review process but prior to a funding commitment	Prior to trial funding, further consideration of the trial design, biostatistics and research methods are needed to enhance understanding of trial informativeness and reduce research waste [47]
2.12. Improve the completeness of trial protocols by enhancing patient‐reported outcome (PRO) data	The PRO content of trials is often suboptimal despite providing valuable evidence to inform shared decision making, labelling claims, clinical guidance and health policy; use of supporting guidelines (e.g. SPIRIT‐PRO) can provide recommendations for items that should be included in protocols in which PROs are a primary or key secondary outcome [48]
2.13. Implement a ‘quality‐by‐design’ approach to clinical trials	A ‘quality‐by‐design’ approach enables organisations to prioritise the most critical determinants of a trial's quality, identify non‐essential activities that can be eliminated to streamline trial conduct and oversight, and formulate appropriate plans to define, avoid, mitigate, monitor, and address important errors [49]
**3.** ***Feasibility: the trial must be demonstrably feasible (e.g., it must have a realistic plan for recruiting sufficient participants)***	
3.1. Integrate qualitative evidence when assessing the feasibility of a trial	Qualitative research findings can further explore lived experiences of a disease condition, such as working within a specific healthcare system, and other barriers and enablers to participation within a trial [16]
3.2. If appropriate, consider a pilot or feasibility study to avoid research waste and de‐risk funding investment(s)	Feasibility studies were found to be potentially useful in assessing whether a more expensive, large‐scale trial was merited [50]; feasibility studies of pragmatic trials may differ, and should consider feasibility objectives specifically relevant to areas of uncertainty for pragmatic trials [51, 52]
3.3. Utilise the knowledge of local health professionals to better inform feasibility or pilot studies	Integration of local health professionals within the trial process can further contextualise issues of recruitment and retention, and help to ensure that the required sample size can be reached [33]
3.4. Include community members in participant recruitment and retention strategies	Concepts of community will vary by global context, but may be particularly relevant in low‐resource settings when considering trial feasibility; community members can provide valuable feedback within retention strategies such as best practices for communication with potential trial participants [33]
**4.** ***Integrity: the trial must be conducted and analysed in a scientifically valid manner that is faithful to the design***	
4.1. Further external information about the treatment effect should be used to inform aspects of the trial analysis	Evidence synthesis of external resources can better inform aspects of trial analysis including further understanding of the treatment effect (e.g., through meta‐analyses, or indirect comparisons), and reduction in bias in the presentation of analysis and trial results [15]
4.2. Further development of skills‐based training to ensure the quality conduct of trials	Good clinical practice (GCP) describes the scientific and ethical considerations involved in the quality conduct of trials; trial teams may benefit from additional training beyond GCP certification in obtaining informed consent, ensuring protocol compliance and protecting participants' health and safety [32, 53]
4.3. Registration of Clinical Trial Units (CTUs) within wider national or international networks	Safeguards capacity for the development and delivery of high‐quality trials by ensuring key competencies for conduct are met within a CTU under the guidance of appropriate expertise [31]
**5.** ***Reporting: systems are in place to ensure timely, complete and accurate reporting***	
5.1. Mandatory requirement to proactively register trials alongside a supporting system of monitoring to ensure compliance with registration, up‐to‐date record keeping and timely publication of results	Publicly specifying details on trial methodology and conduct before enroling participants increases transparency, decreases selective reporting and subsequent publication bias, and ensures an ethical responsibility to publicly report trial results [26, 54]
5.2. Further reporting within appropriate registries	Patient registries and databases can further support clinical research and may be vital to assessing the feasibility of trials for areas such as rare or orphan diseases; reporting should consider the relevance of the trial to future research and contribute to registries as appropriate [38]
5.3 Reporting of health equity considerations	Transparency and completeness within the trial reporting process should consider how the presentation of the intervention and trial results may influence policy making and other decisions for those who are currently underserved by health research and health services [28]

### 3.3 Tools for Improving Trial Informativeness

A key recommendation from the research was the use of ‘tools’ to enhance trial informativeness (e.g., Recommendation 2.1 in Table 2). This review did not seek out tools specifically. However, it became evident that tools form a significant discussion point within the topic of trial informativeness. Tools presented as standalone research within this review, or those used to facilitate active engagement in a wider trial design process, are summarised in Table 3. A total of 23 tools were identified across Zarin and colleagues' conditions of 1) Importance (17%), 2) Design (74%) and 5) Reporting (9%). Additional citations are provided where tools are available through Open Access or other digital platforms.

**Table 3:** Tools to further improve aspects of trial informativeness.

**Supporting tool**	**Description**
**1.** ***Importance: trial hypothesis is likely to inform an important scientific, medical or policy decision***	
Enhancing Quality in Preclinical Data (EQIPD) Quality System [22]	▪ Quality framework and interactive web‐based tool that enables research units to evaluate and fulfil their own quality needs; intended to increase adherence to rigorous, evidence‐based practices in preclinical research through a set of 18 core requirements that can be addressed flexibly according to use‐specific needs
Evidence maps [13]	▪ Described as a systematic organisation and illustration of a broad field or research evidence with the intent to characterise the breadth, depth and methodology of relevant evidence to identify gaps
Multi‐criteria tool for evaluating research proposals [60]	▪ Proposed tool captures four criteria that represent the ‘worth’ or merit of a research project; can be used to formalise a process of ranking to help identify the best proposals for funding
Value of Information (VOI) analyses [17, 18]	▪ VOI analyses calculate the economic value that could be generated by obtaining further information to reduce uncertainty in health economic decision models; suggested as a tool for research prioritisation *and* trial design as it can highlight economically valuable avenues for future research
**2.** ***Design: trial methods are likely to provide meaningful evidence related to the study hypothesis***	
American Society of Oncology Clinical (ASCO) Trial Workload Assessment Tool [30]	▪ Uses a 4‐point protocol acuity rating scale which distinguishes trials on a continuum from less complex trials to very complex trials; the assessment process can be further supported by the ASCO Research Program Quality Assessment Tool [61]
A Study Pragmatic‐Explanatory Characterisation Tool‐Rating (ASPECT‐R) [56]	▪ Adaptation of current tools (including PRECIS [62]) for design support and post hoc evaluation of trials within a framework that is either more explanatory or pragmatic; intended to improve consistency of use and interpretation across raters
Clinical Trials Transformation Initiative (CTTI) ‘quality‐by‐design’ toolkit [49]	▪ Online toolkit is available to support ‘quality‐by‐design’ in practice, including further educational and dissemination resources, and resources to further implement a quality‐by‐design approach within a clinical trial
Data mining [40]	▪ Use of data mining (contrast mining) to provide explainable and potentially modifiable recommendations for new trial designs
Factors to inform the most likely trial design (superiority, equivalence and non‐inferiority) [46]	▪ A 19‐item list of factors that aims to assist decision‐making when a study is being planned that mirrors the population, intervention, comparison, outcome (PICO) format and the estimand framework
GetReal Trial Tool [35]	▪ Allows users to assess the impact of design choices on generalisability to routine clinical practice, while considering risk of bias, precision, acceptability and operational feasibility
Global Health Research Process Map [33]	▪ Interactive web‐based tool providing pragmatic, globally applicable advice to the planning of rigorous health research studies with an emphasis on clinical trials
Health equity design framework [28]	▪ Conceptual framework that may be used to design health equity‐relevant randomised trials (as well as other study types) and to identify health equity‐relevant studies that contribute to an evidence base that improves overall health and health equity
Internet‐based crowdsourcing platform [63]	▪ Through crowdsourcing via an internet‐based platform, the intellectual and creative capacity of a large number of researchers, clinicians and patients was used to improve the clinical design process
Machine learning [39]	▪ Prediction of clinical trial success (early termination) using machine learning algorithms based on previous data collected from clinical studies; provides further insights on features of trial design that may contribute to early termination
Maturity model for the scientific review of clinical trial designs [47]	▪ Maturity model including 11 process areas and 5 maturity levels; each of the 55 process area levels are populated with descriptions on a continuum toward an optimal state to improve trial protocols in the areas of risk of failure or uninformativeness
Patient Motivation Pyramid [24]	▪ Based on Maslow's theory of human motivation as a tool to identify patient needs; used to create a comprehensive overview of options to implement a patient‐centric trial design
PONTE Authoring Tool (PAT) [57]	▪ Flexible trial design tool that utilises clinical care information system, clinical research information systems, and drug and disease knowledge databases alongside advanced data mining techniques and enhanced learning algorithms
Pragmatic–Explanatory Continuum Indicator Summary (PRECIS‐2) [34]	▪ Framework to guide study teams to *prospectively* consider the pragmatic or explanatory nature of their trial across 9 domains
Protocol Ethics Tool Kit [43]	▪ For use by investigators or trial sponsors/funders to develop a dedicated section for ethics within a trial protocol to improve the consistency and transparency between clinical trial protocols and research ethics committee reviews
SPIRIT‐PRO Extension [48]	▪ Reporting guideline for clinical trial protocols in which patient‐reported outcomes (PROs) are a primary or key secondary outcome
Trial Innovation Network (TIN) toolbox [37]	▪ Resources to support the engagement, recruitment and retention activities for multi‐site clinical trials, including best evidence‐based recruitment information
**5.** ***Reporting: systems are in place to ensure timely, complete and accurate reporting***	
Checklist for reporting benefit–risk method [46]	▪ When a benefit‐risk method is intended to be or has been used, it is important to include all relevant information via the proposed checklist when reporting the trial design or the results of the trial
Registry design checklist [38]	▪ Type of information that should be included in a rare disease trial registry to inform clinical trial design and reporting

### 3.4 Applicability of Findings to Processes of Pre‐Funding Peer Review

Approximately 10% of the research we reviewed broadly discussed pre‐funding peer review systems. There was an emphasis on the need for higher‐quality peer review processes to ensure that only the most promising and relevant research is funded [25, 47]. The need for relevant expertise was also highlighted, including input from those who had previously undertaken or contributed to a successful process of peer review [31, 33]. While many findings from Table 2 may be applicable to pre‐funding peer review, more research is needed before additional recommendations can be made.

## 4 Discussion

This rapid review sought to provide insights on actionable trial design processes that could improve the informativeness of trials as described by Zarin and colleagues [8, 9]. The concept of an ‘informative’ trial is still evolving and while research waste can occur at any stage, an informative trial seeks to eliminate waste altogether. In 2023, the World Health Organisation outlined draft best practices for clinical trials where ‘good trials’ have been described as those that are ‘reliably informative, ethical and efficient and answer scientifically important questions relevant to the populations they are intended to benefit’ [55]. Zarin and colleagues' conditions for informativeness provide some of the first measures to define and evaluate what makes a trial ‘reliably informative’.

Where this rapid review differs from the current literature is in the synthesis of *process* and how standalone recommendations, exemplars and tools are reflective of a need for wider top‐level change. For instance, within Zarin and colleagues' first condition of 1) Importance, most research emphasised the need for a formal process to prioritise research questions that fill current evidence gaps. This was underscored by examples showcasing the value of differing evidence syntheses, the need for economic analyses, and how the priorities of stakeholders may influence agenda setting. These examples highlight how poorly defined trial hypotheses contribute to research waste, and how the duplication of effort in funding previously researched or similarly identified studies explicitly fails to further informativeness.

Throughout the recommended processes or actions to improve informativeness as relevant to Zarin and colleagues' second condition, 2) Design, was a need to ensure trial methods were fit‐for‐purpose, that is, the methods and design choices are appropriate and reflect the intended aims of the trial. In considering the generalisability of trial results, several authors discussed the benefits of a pragmatic trial design where the effectiveness of the intervention is evaluated under conditions close to those in which the intervention would be used routinely, should it prove effective [33, 34, 35, 51, 56]. However, any given trial may contain both pragmatic and more explanatory elements [34, 35, 56]. This is where design tools, such as those captured in Table 3, are of benefit when considering trial informativeness. The most referenced tool throughout the review was PRECIS‐2, which aims to help those designing trials to proactively consider where they would like their trial to be on the pragmatic/explanatory continuum [34].

While validated tools such as PRECIS‐2 are the current standard, the review also captured several conceptual and applied uses of artificial intelligence [39, 40, 57]. These examples largely utilised current knowledge of successful trials to inform future design practices and are likely to be a key area of further development given the rapid rate of digital transformation across all areas of health and social care. Digital advances also include Open Science (OS) practices, which can support both an initial design process and the ongoing conduct and reporting of a trial [25]. OS practices such as pre‐registering prospective projects on online platforms or submitting a registered report further support trial informativeness by allowing others to access relevant information, including public access to ongoing research. Studies have also shown that researchers who share data ‘produced findings with fewer errors, thereby suggesting more diligence in scientific practice and reporting’ [25].

Most of the research captured by this review was from either the USA (28.6%) or the UK (21.4%). However, aspects of fit‐for‐purpose trial design for low‐resource settings were also noted in the context of sub‐Saharan Africa [21, 33, 36]. In these instances, authors reported a disconnect between the priorities and expectations of outside funders and the realities of designing and implementing trials in low‐resource settings where healthcare resources and systems may not meet with rich‐world standards. For a trial to be informative in a low‐resource setting, the design process must consider the regional context, including culture [36]. Studies found that better prospective integration of the local community, healthcare professionals, and trial staff led to better‐quality protocols requiring fewer amendments and overall improved trial feasibility [21, 33, 36].

The importance of a realistic plan for recruiting sufficient trial participants is reflected in Zarin and colleagues' third condition for informativeness, 3) Feasibility. This includes the need for improved infrastructure to track and address recruitment issues [8]. Feasibility as described by Zarin and colleagues also further highlights how the conditions of informativeness may intersect. For example, feasibility studies were suggested as a means to ensure trial design decisions match the intended decision‐making purpose of the trial results (i.e., 2) Design) and can adequately generate meaningful evidence related to the study hypothesis (i.e., 1) Importance) [50, 51]. Communication was also a key aspect in understanding how feasibility could be improved, such as including relevant community members and health experts in the design of recruitment and retention strategies [23, 33].

Zarin and colleagues' fourth condition for informativeness, 4) Integrity, reflected processes or actionable change at both the level of the individual as well as wider considerations of a Clinical Trial Unit or associated trial network [31, 53]. This review found that individuals and trial teams may benefit from additional practical, skills‐based training to further contextualise protocol compliance and aspects of participant health and safety, such as obtaining informed consent [32, 53]. The importance of expertise in multi‐centre or multi‐site trials where the success of the trial relies on multiple individuals and groups working collaboratively to achieve differing conditions of informativeness was also noted [36, 37].

The final condition for informativeness, 5) Reporting, focused on the need to proactively register trials before enroling participants to enhance transparency and decrease selective reporting and subsequent publication bias [26, 54]. Proactively registering trials also fulfils ethical obligations as outlined by the Declaration of Helsinki which states that ‘every research study involving human subjects must be registered in a publicly accessible database before recruitment of the first subject’ [7]. This is seconded by the International Committee of Medical Journal Editors who advise consideration of trials for publication only if registered as appropriate [58]. One study found that less than half (42.9%) of the largest public and philanthropic medical research funders in Europe monitored whether trials were registered, further supporting the need for increased accountability on the part of funders or sponsors to ensure appropriate trial reporting [26].

As this was a rapid review of global processes that may improve trial informativeness, we sought to be resource‐efficient by omitting elements of a traditional systematic review, such as searching multiple sources or incorporating grey literature. It is worth noting that this rapid review is the first part of a body of work that will also include an analysis of grey literature, including funder guidance for applicants, as well as a global interview study with relevant parties within trials research.

We recognise that the concept of ‘informativeness’ is not yet globally widespread in the lexicon of trials research and that our rapid review methods may have omitted further work, such as the pre‐print by Burford and colleagues who have suggested an additional list of recommendations to enhance the informativeness of global health trials [59]. However, by targeting peer‐reviewed, published resources, and considering these alongside the work of Zarin and colleagues, we have identified processes or other actions that trialists can implement to improve the informativeness of trials. This is complemented by a list of trial design tools, many of which are available via Open Access resources or other digital platforms.

## 5 Conclusion

While RCTs are considered the ‘gold standard’ of clinical research, many are uninformative and contribute to research waste. The insights from global research captured by this review provide a series of recommendations aimed at improving trial informativeness in line with the five conditions proposed by Zarin and colleagues. While not explicitly sought as part of this review, the development and use of trial design ‘tools’ was found to be a key recommendation for improving informativeness. Further research is needed on how trial design processes can better support pre‐funding peer review, which would increase the likelihood of producing informative trials.

## Author Contributions

**Sarah R. Prowse:** conceptualisation, methodology, formal analysis, investigation, resources, writing – original draft, project administration. **Shaun Treweek:** conceptualisation, methodology, validation, writing – review and editing, supervision, funding acquisition. **Miriam Brazzelli:** conceptualisation, methodology, validation, writing – review and editing, supervision. **Hanne Bruhn:** conceptualisation, methodology, validation, writing – review and editing.

## Conflicts of Interest

Shaun Treweek is a co‐author of 2 of the 42 included studies [34, 45].

## Supporting information

Supporting Material 1 541229 STRATEGY 20240617.

Supporting Material 2 Data extraction forms.

Supporting Material 3 JBI Checklists.

## Data Availability

The data underlying this article are available in the article and in its online Supporting Material.
