# Supplementary material for: Improving Trial Informativeness: A Rapid Review of Global Research on How to Ensure Trials Are Useful
Source: J Eval Clin Pract. 2025 Jun 11;31(4):e70147. doi: 10.1111/jep.70147 (PMC12158544; doi:10.1111/jep.70147)
Supplement: Supplementary file 1 — Supporting Material 1 541229 STRATEGY 20240617. [file JEP-31-0-s002.pdf]

Ovid MEDLINE(R) and Epub Ahead of Print, In-Process, In-Data-Review & Other Non-Indexed Citations, Daily and Versions <1946 to May 08, 2024>

1 exp *biomedical research/

2 exp *Clinical studies as topic/

3 *Research Support as Topic/

4 1 or 2 or 3

5 *research design/

6 4 and 5

7 ((increas* or improv* or encourag* or support* or promot* or influen*) adj4 (trial? adj3 (quality or conduct or method* or practice* or design? or importance or integrity))).ti,ab,kw.

8 ((increas* or improv* or encourag* or support* or promot* or influen*) adj4 ((trial? or data*4) adj4 transparen*)).ti,ab,kw.

9 ((increas* or improv* or encourag* or support* or promot* or influen*) adj4 (reproducib* adj4 (trial? or data*4 or result* or finding* or conclusion* or method* or design?))).ti,ab,kw.

10 ((decreas* or minimis* or reduc* or discourag* or prevent*) adj4 (research adj2 (waste*1 or wastage))).ti,ab,kw.

11 ((decreas* or minimis* or reduc* or discourag* or prevent*) adj4 (unpublished adj3 (data*4 or research))).ti,ab,kw.

12 ((decreas* or minimis* or reduc* or discourag* or prevent*) adj4 ((poor* or inadequate*) adj3 (design? or method*))).ti,ab,kw.

13 ((decreas* or minimis* or reduc* or discourag* or prevent*) adj4 (trial? and (design? adj2 (flaw* or limitation* or choice*1)))).ti,ab,kw.

14 ((increas* or improv* or encourag* or support* or promot* or influen*) adj4 ((journal* or editor* or publisher* or funding or funder*1 or legal) adj3 (complian* or non-complian* or adher* or policy or policies or guideline* or guidance))).ti,ab,kw.

15 ((increas* or improv* or encourag* or support* or promot* or influen*) adj4 (open adj2 (research or data*4 or science or practice*))).ti,ab,kw.

16 ((increas* or improv* or encourag* or support* or promot* or influen*) adj4 informativeness).tw,kf.

17 or/7-16

18 (((review* or check* or evaluat* or assess* or examin*) adj5 (design? or early) adj5 trial?) not (systematic adj3 review)).tw.

19 5 and 18

19 6 or 18 or 19

20 limit 19 to yr="2014 -Current" 9137
