# Supplementary material for: Improving Trial Informativeness: A Rapid Review of Global Research on How to Ensure Trials Are Useful
Source: J Eval Clin Pract. 2025 Jun 11;31(4):e70147. doi: 10.1111/jep.70147 (PMC12158544; doi:10.1111/jep.70147)
Supplement: Supplementary file 2 — Supporting Material 2 Data extraction forms. [file JEP-31-0-s003.pdf]

Section 1: Publication details	
Author(s) or Organization(s)	Alahdab & Murad
Title of publication	Evidence maps: a tool to guide research agenda setting
Link to publication	https://doi.org/10.1136/bmjebm-2018-111137
Year of publication	2018
Geographic setting of the research (or, geographic affiliations of publishing authors)	USA
Funder and/or sponsor of the research	The authors have not declared a specific grant for this research from any funding agency in the public, commercial or not-for-profit sectors
**Section 2: Intervention details**	
Type of intervention or proposed intervention to improve trial informativeness	Evidence maps
Role of the intervention or proposed intervention within the trials research pathway	Research agenda setting
If stated, domain of study (E.g., research waste, misconduct, feasibility...)	Evidence synthesis; research waste
Most relevant to Zarin et al? Or, other...	(1) Importance: trial hypothesis is likely to inform an important scientific, medical, or policy decision
If applicable, population (or target population for the intervention)	Clinical investigators
If applicable, method(s) used to develop the intervention	See: Online Supplementary Box 1
If applicable, sample size used to develop the intervention	N/A
If applicable, further details on the design or function of the proposed or actualized intervention	See: Online Supplementary Box 1
If applicable, details of the evaluative process for the proposed or actualized intervention	N/A
**Section 3: Outcomes**	
If applicable, outcomes measured (or anticipated outcomes)	N/A
Author's conclusion as verbatim text:	
Research waste remains a problem caused by a number of reasons. Asking the wrong research questions and ignoring the existing evidence are possible preventable reasons. Evidence maps are tools that may aid in guiding clinical investigators and help in agenda setting of future research.	
(Optional) References to other mentioned tools or studies of interest	Consolidated Standards of Reporting Trials (CONSORT)
**Section 4: Additional notes**	
Additional notes	N/A

**Section 1: Publication details**	
Author(s) or Organization(s)	Al-Durra et al.
Title of publication	Prospective registration and reporting of trial number in randomised clinical trials: global cross sectional study of the adoption of ICMJE and Declaration of Helsinki recommendations
Link to publication	https://doi.org/10.1136/bmj.m982
Year of publication	2020
Geographic setting of the research (or, geographic affiliations of publishing authors)	Global (geographic scope); Canada (authors)
Funder and/or sponsor of the research	None received
**Section 2: Intervention details**	
Type of intervention or proposed intervention to improve trial informativeness	Prospective registration and inclusion of the trial registration number in published randomised controlled trials
Role of the intervention or proposed intervention within the trials research pathway	Reporting (prospective)
If stated, domain of study per the authors (E.g., research waste, misconduct, feasibility...)	Compliance
Most relevant to Zarin et al? Or, other...	(5) Reporting: systems are in place to ensure timely, complete and accurate reporting
If applicable, population (or target population for the intervention)	Trial investigators
If applicable, method(s) used to develop the intervention	Cross-sectional analysis of published studies
If applicable, sample size used to develop the intervention	10,500 manuscripts published in 2105 journals indexed in PubMed and registered across the WHO trial registries
If applicable, further details on the design or function of the proposed or actualized intervention	Empirical appraisal of the current state of compliance with the ICMJE recommendation 10 years after the adoption of the seventh revision of the Declaration of Helsinki; investigated compliance with the inclusion of the trial registration number in published RCTs and compliance with the prospective registration of RCTs across all the WHO trial registries.
If applicable, details of the evaluative process for the proposed or actualized intervention	N/A
**Section 3: Outcomes**	
If applicable, outcomes measured (or anticipated outcomes)	Compliance with prospective trial registration; selective registration bias
Author's conclusion as verbatim text:	
Compliance with including the TRN in published trials was 71.2% overall and significantly higher (95.5%) for papers published in an ICMJE member journal. These journals should be commended for adherence to their mandate and driving the adoption within the broader community of scholarly medical journals.<br><br>*We found low compliance with prospective trial registration, as defined by the ICMJE (ie, when a trial is registered before the enrolment of the first participant) at 41.7% overall and 61.3% for trials published in one of the ICMJE member journals. The empirically low adoption, even within the ICMJE member journals, 10 years after the seventh revision of the Declaration of Helsinki, questions the adaptability and viability of this recommendation. We detected a new form of bias, referred to as selective registration bias, with a statistically significant high proportion (85.2%) of investigators registering their trials retrospectively within one year of submission to a journal.*	

Within the cohort of trials that were registered retrospectively and published in one of the ICMJE member journals, we measured author compliance with publishing a statement justifying the late registration and the reasons why journal editors accept submissions of retrospectively registered trials. No published editor's statement was identified, and only 2.8% of the authors included a statement justifying the delayed registration. Reasons for late registration were lack of awareness, error of omission, or because the registration process took longer than anticipated. Some authors emphasised that late registration was completed before any data analysis or collection.<br><br>*This assessment of the adoption of the ICMJE and WHO recommendations for the prospective registration of clinical trials should help to inform the future development of publication and registration guidelines of RCTs for researchers, policy makers, and journal editors.*	
(Optional) References to other mentioned tools or studies of interest	N/A
**Section 4: Additional notes**	
Additional notes	N/A

**Section 1: Publication details**	
Author(s) or Organization(s)	Alphs & Bossie
Title of publication	ASPECT-R-A Tool to Rate the Pragmatic and Explanatory Characteristics of a Clinical Trial Design
Link to publication	https://pubmed.ncbi.nlm.nih.gov/27413583
Year of publication	2016
Geographic setting of the research (or, geographic affiliations of publishing authors)	USA
Funder and/or sponsor of the research	Janssen Scientific Affairs
**Section 2: Intervention details**	
Type of intervention or proposed intervention to improve trial informativeness	Adaptation of PRECIS and Pragmascope instruments; A Study Pragmatic-Explanatory Characterization Tool-Rating (ASPECT-R)
Role of the intervention or proposed intervention within the trials research pathway	Design support and post-hoc evaluation of clinical trials
If stated, domain of study per the authors (E.g., research waste, misconduct, feasibility...)	Trial design
Most relevant to Zarin et al? Or, other...	(2) Design: trial methods are likely to provide meaningful evidence related to the study hypothesis (also see Zarin et al; (3) Feasibility, and (4) Integrity)
If applicable, population (or target population for the intervention)	Researchers, clinicians, healthcare providers, and policymakers
If applicable, method(s) used to develop the intervention	Adaptation of PRECIS and Pragmascope instruments
If applicable, sample size used to develop the intervention	N/A
If applicable, further details on the design or function of the proposed or actualized intervention	ASPECT-R consists of six domains deemed important for characterizing the explanatory to pragmatic spectrum of study designs; domain ratings are based upon the rater's review of the published manuscript and any additional information that reflects the study design and trial methodology.
If applicable, details of the evaluative process for the proposed or actualized intervention	N/A
**Section 3: Outcomes**	
If applicable, outcomes measured (or anticipated outcomes)	See: Appendix 1 (ASPECT-R worksheets)
Author's conclusion as verbatim text:	
This new tool, ASPECT-R, should provide a reliable, objective way to rate studies along the explanatory-pragmatic spectrum that will better support trial design and facilitate interpretation of completed trials. The complete ASPECT-R tool and guide materials can be accessed online by clicking or visiting this link: http://innovationscns.com/aspect-r-tool-and-training-materials/	
(Optional) References to other mentioned tools or studies of interest	PRECIS; Pragmascope
**Section 4: Additional notes**	
Additional notes	Link to training materials defunct; worksheets can be accessed via the published Appendices.

Section 1: Publication details	
Author(s) or Organization(s)	Bespalov et al.
Title of publication	Introduction to the EQIPD quality system
Link to publication	https://doi.org/10.7554/elife.63294
Year of publication	2021
Geographic setting of the research (or, geographic affiliations of publishing authors)	Europe (broadly)
Funder and/or sponsor of the research	Innovative Medicines Initiative 2 Joint Undertaking (European Union's Horizon 2020 research and innovation programme and EFPIA)
**Section 2: Intervention details**	
Type of intervention or proposed intervention to improve trial informativeness	Preclinical research quality system (EQIPD Quality System)
Role of the intervention or proposed intervention within the trials research pathway	Generation of preclinical data for use in trial design; option for further processes of trial management and reporting
If stated, domain of study per the authors (E.g., research waste, misconduct, feasibility...)	Quality management
Most relevant to Zarin et al? Or, other...	Focus on (2) Design: trial methods are likely to provide meaningful evidence related to the study hypothesis (also see Zarin et al; (4) Integrity and (5) Reporting)
If applicable, population (or target population for the intervention)	Scientists directly involved in the research, but also their funders, sponsors, publishers, research tool manufacturers, and collaboration partners such as peers in a multi-site research project
If applicable, method(s) used to develop the intervention	Developed by the EQIPD consortium (29 institutions across 8 countries, plus additional stakeholders):<br>• Phase 1: systematic review, inventory of current practice, review of existing quality systems<br>• Phase 2: working group and Delphi study<br>• Phase 3: framework development and pilot of quality system
If applicable, sample size used to develop the intervention	• Phase 1: inventory of current practices; interviews with 70 consortium members<br>• Phase 2: working group of 20 EQIPD consortium members<br>• Phase 3: Quality system implemented at four independent research sites
If applicable, further details on the design or function of the proposed or actualized intervention	See: Table 3 (EQIPD Quality System key principles and features)
If applicable, details of the evaluative process for the proposed or actualized intervention	See: Appendices
**Section 3: Outcomes**	
If applicable, outcomes measured (or anticipated outcomes)	N/A
Author's conclusion as verbatim text:	
The EQIPD Quality System proposes guidance on expectations for quality-related measures, defines criteria for adequate processes (i.e. performance standards) and provides examples of how such measures can be developed and implemented. However, it does not prescribe any pre-determined solutions. EQIPD has also developed tools (for optional use) to support users in implementing the system and assessment services for those research units that successfully implement the quality system and seek formal accreditation. Building upon the feedback from users and continuous	

improvement, a sustainable EQIPD Quality System will ultimately serve the entire community of scientists conducting non-regulated preclinical research, by helping them generate reliable data that are fit for their intended use.	
(Optional) References to other mentioned tools or studies of interest	N/A
**Section 4: Additional notes**	
Additional notes	Authors further promote: http://www.eqipd.online/ (appears defunct).

Section 1: Publication details	
Author(s) or Organization(s)	Brewster et al.
Title of publication	Introduction to the development of complex interventions
Link to publication	https://doi.org/10.1136/postgradmedj-2021-139766
Year of publication	2021
Geographic setting of the research (or, geographic affiliations of publishing authors)	UK (England)
Funder and/or sponsor of the research	The authors have not declared a specific grant for this research from any funding agency in the public, commercial or not-for-profit sectors
**Section 2: Intervention details**	
Type of intervention or proposed intervention to improve trial informativeness	Medical Research Council's guidance and framework (UK MRC Framework)
Role of the intervention or proposed intervention within the trials research pathway	The UK MRC Framework is a systematic and phased approach to intervention design, while building evidence and testing theory before implementing an intervention or conducting a trial
If stated, domain of study per the authors (E.g., research waste, misconduct, feasibility...)	Application of the UK MRC Framework and applicable tools to reduce research waste
Most relevant to Zarin et al? Or, other...	Focus on (2) Design: trial methods are likely to provide meaningful evidence related to the study hypothesis (also see Zarin et al; (3) Feasibility, (4) Integrity, and (5) Reporting)
If applicable, population (or target population for the intervention)	Intervention developers
If applicable, method(s) used to develop the intervention	N/A; overview of pre-existing MRC Framework
If applicable, sample size used to develop the intervention	N/A; overview of pre-existing MRC Framework
If applicable, further details on the design or function of the proposed or actualized intervention	Four stages of the 2008 MRC Framework:<br>1. Development of the complex intervention: reviewing the evidence base, identifying and developing theory, and modelling processes and outcomes.<br>2. Feasibility and piloting: testing procedures, estimating retention and determining sample size.<br>3. Evaluation: assessing effectiveness, understanding the change process and evaluating cost-effectiveness.<br>4. Implementation: dissemination, surveillance, monitoring and long-term follow-up.
If applicable, details of the evaluative process for the proposed or actualized intervention	N/A
**Section 3: Outcomes**	
If applicable, outcomes measured (or anticipated outcomes)	N/A
Author's conclusion as verbatim text:	
With the rising number of people living with multimorbidity, healthcare interventions to support these individuals must be developed with an appreciation for its complexity. Although the MRC provides guidance on developing and evaluating complex interventions, a number of frameworks exist to help provide more structure during the early development phase. Two widely used frameworks used in behaviour change interventions include the BCW and IM. In some instances, the person-based approach is used to supplement these models, but to date, this has typically been in relation to	

technology interventions. The BCW and IM are well-cited tools in behaviour change interventions for long-term health conditions. Although they share many similarities, they also celebrate key differences, some of which have been described in this article.<br><br>*By applying the MRC framework, with the addition of appropriate tools to supplement the development phase, it is hoped that complex interventions to address multimorbidity will result in less research 'waste'. Following a detailed and explicit process, as outlined in this article, can help with the later identification of 'active' and reproducible components of an intervention, so that aspects of it may be successfully transferred to other contexts.*	
(Optional) References to other mentioned tools or studies of interest	Behaviour Change Wheel and Intervention Mapping
**Section 4: Additional notes**	
Additional notes	Secondary focus on behaviour change interventions and intersection with the MRC Framework.

Section 1: Publication details	
Author(s) or Organization(s)	Bruckner et al.
Title of publication	Adoption of World Health Organization Best Practices in Clinical Trial Transparency Among European Medical Research Funder Policies
Link to publication	https://doi.org/10.1001/jamanetworkopen.2022.22378
Year of publication	2022
Geographic setting of the research (or, geographic affiliations of publishing authors)	Europe (Germany, Sweden, The Netherlands); UK (England)
Funder and/or sponsor of the research	Not stated
**Section 2: Intervention details**	
Type of intervention or proposed intervention to improve trial informativeness	Reporting standards
Role of the intervention or proposed intervention within the trials research pathway	Prospective trial reporting; further monitoring and proposed sanctions
If stated, domain of study per the authors (E.g., research waste, misconduct, feasibility...)	Reporting (to reduce research waste and publication bias)
Most relevant to Zarin et al? Or, other...	(5) Reporting: systems are in place to ensure timely, complete and accurate reporting
If applicable, population (or target population for the intervention)	Research funders
If applicable, method(s) used to develop the intervention	Cross-sectional analysis based on independent assessments of publicly available funder documentation and validation of results with funders
If applicable, sample size used to develop the intervention	21 of the largest non-multilateral public and philanthropic funders in Europe
If applicable, further details on the design or function of the proposed or actualized intervention	N/A (see evaluative process below)
If applicable, details of the evaluative process for the proposed or actualized intervention	Scoring of funders using an 11-item assessment tool based on WHO best practice benchmarks, grouped into 4 broad categories: trial registries, academic publication, monitoring, and sanctions. Funder references to reporting standards were also captured.
**Section 3: Outcomes**	
If applicable, outcomes measured (or anticipated outcomes)	The primary outcome was funder adoption or non-adoption of 11 policy and monitoring measures to reduce research waste and publication bias as set out by WHO best practices. The secondary outcomes were whether and how funder policies referred to reporting standards. Outcomes were preregistered after a pilot phase that used the same outcome measures.
Author's conclusion as verbatim text:	
This study found that many European medical research funder policy and monitoring measures fell short of WHO best practices. These findings suggest that funders worldwide may need to identify and address gaps in policies and processes.	
(Optional) References to other mentioned tools or studies of interest	N/A
**Section 4: Additional notes**	
Additional notes	N/A

Section 1: Publication details	
Author(s) or Organization(s)	Calvert et al.
Title of publication	Guidelines for Inclusion of Patient-Reported Outcomes in Clinical Trial Protocols: The SPIRIT-PRO Extension
Link to publication	https://doi.org/10.1001/jama.2017.21903
Year of publication	2018
Geographic setting of the research (or, geographic affiliations of publishing authors)	UK (England); Australia; Canada
Funder and/or sponsor of the research	Macmillan Cancer Support; University of Birmingham
**Section 2: Intervention details**	
Type of intervention or proposed intervention to improve trial informativeness	International, consensus-based, patient-reported outcome (PRO)-specific protocol guidance
Role of the intervention or proposed intervention within the trials research pathway	Inclusion of PRO content at the stage of clinical trial protocol development
If stated, domain of study per the authors (E.g., research waste, misconduct, feasibility...)	Protocol development/guidance
Most relevant to Zarin et al? Or, other...	Focus on (2) Design: trial methods are likely to provide meaningful evidence related to the study hypothesis (also see Zarin et al; (4) Integrity, and (5) Reporting)
If applicable, population (or target population for the intervention)	Clinical trial research personnel, PRO methodologists, health economists, psychometricians, patient advocates, funders, industry representatives, journal editors, policy makers, ethicists, and researchers responsible for evidence synthesis
If applicable, method(s) used to develop the intervention	The SPIRIT-PRO Extension was developed following the Enhancing Quality and Transparency of Health Research (EQUATOR) Network's methodological framework for guideline development.
If applicable, sample size used to develop the intervention	The final wording of the SPIRIT-PRO Extension was agreed on at a consensus meeting (n = 29 participants) and reviewed by external group of experts during a consultation period.
If applicable, further details on the design or function of the proposed or actualized intervention	The final SPIRIT-PRO Extension recommends that, in conjunction with existing SPIRIT 2013 items, 16 items (11 extensions and 5 elaborations) should be routinely addressed in all clinical trial protocols in which PROs are a primary or key secondary outcome.
If applicable, details of the evaluative process for the proposed or actualized intervention	N/A
**Section 3: Outcomes**	
If applicable, outcomes measured (or anticipated outcomes)	N/A
Author's conclusion as verbatim text:	
The SPIRIT-PRO guidelines provide recommendations for items that should be addressed and included in clinical trial protocols in which PROs are a primary or key secondary outcome. Improved design of clinical trials including PROs could help ensure high-quality data that may inform patient-centered care.	
(Optional) References to other mentioned tools or studies of interest	SPIRIT (Standard Protocol Items: Recommendations for Interventional Trials)
**Section 4: Additional notes**	
Additional notes	N/A

Section 1: Publication details	
Author(s) or Organization(s)	Chan et al.
Title of publication	Pilot and feasibility studies for pragmatic trials have unique considerations and areas of uncertainty
Link to publication	https://doi.org/10.1016/j.jclinepi.2021.06.029
Year of publication	2021
Geographic setting of the research (or, geographic affiliations of publishing authors)	UK; Canada
Funder and/or sponsor of the research	Canadian Institutes of Health Research; National Institute of Aging (NIA) of the National Institutes of Health
**Section 2: Intervention details**	
Type of intervention or proposed intervention to improve trial informativeness	Identification of unique areas of uncertainty that are relevant to planning a pragmatic trial
Role of the intervention or proposed intervention within the trials research pathway	Trial design
If stated, domain of study per the authors (E.g., research waste, misconduct, feasibility...)	Feasibility
Most relevant to Zarin et al? Or, other...	Focus on (3) Feasibility: the trial is likely to be feasible (also see Zarin et al; (2) Design)
If applicable, population (or target population for the intervention)	Researchers planning a feasibility study in advance of a pragmatic trial
If applicable, method(s) used to develop the intervention	Use of PRECIS-2 to identify initial potential areas of uncertainty
If applicable, sample size used to develop the intervention	N/A
If applicable, further details on the design or function of the proposed or actualized intervention	Two additional domains identified outside of PRECIS-2; intervention development and research ethics
If applicable, details of the evaluative process for the proposed or actualized intervention	N/A
**Section 3: Outcomes**	
If applicable, outcomes measured (or anticipated outcomes)	N/A
Author's conclusion as verbatim text:	
Researchers planning a feasibility study in advance of a pragmatic trial should consider feasibility objectives specifically relevant to areas of uncertainty for pragmatic trials.	
(Optional) References to other mentioned tools or studies of interest	PRECIS-2
**Section 4: Additional notes**	
Additional notes	N/A

**Section 1: Publication details**	
Author(s) or Organization(s)	Chang et al.
Title of publication	Understanding common key indicators of successful and unsuccessful cancer drug trials using a contrast mining framework on ClinicalTrials.gov
Link to publication	https://doi.org/10.1016/j.jbi.2023.104321
Year of publication	2023
Geographic setting of the research (or, geographic affiliations of publishing authors)	USA
Funder and/or sponsor of the research	Not stated
**Section 2: Intervention details**	
Type of intervention or proposed intervention to improve trial informativeness	Contrast mining framework (data mining)
Role of the intervention or proposed intervention within the trials research pathway	Trial design
If stated, domain of study per the authors (E.g., research waste, misconduct, feasibility...)	Research waste
Most relevant to Zarin et al? Or, other...	(1) Importance: trial hypothesis is likely to inform an important scientific, medical, or policy decision, and equally (2) Design: trial methods are likely to provide meaningful evidence related to the study hypothesis
If applicable, population (or target population for the intervention)	Clinical trial developers
If applicable, method(s) used to develop the intervention	First computationally classified cancer drug trials into successful and unsuccessful cases and then utilized natural language processing to extract eligibility criteria information from the trial documents. To provide explainable and potentially modifiable recommendations for new trial design, contrast mining was applied to discover highly contrasted patterns with a significant difference in prevalence between successful (completion with advancement to the next phase) and unsuccessful (suspended, withdrawn, or terminated) groups.
If applicable, sample size used to develop the intervention	49,032 trial documents on ClinicalTrials.gov
If applicable, further details on the design or function of the proposed or actualized intervention	The proposed framework extracts highly contrasted patterns of combinations of trial characteristics with significant difference in prevalence between the successful and unsuccessful trials. Such contrast patterns can be combinations of trial characteristics from multiple aspects which indicates association between the trial characteristics and represents their multifactorial association with trial outcomes.
If applicable, details of the evaluative process for the proposed or actualized intervention	N/A
**Section 3: Outcomes**	
If applicable, outcomes measured (or anticipated outcomes)	Contrast pattern mining can identify explainable patterns which are potentially actionable for trial investigators to implement; the authors also propose how the work could impact modernization efforts for ClinicalTrials.gov, particularly for cancer treatments.
Author's conclusion as verbatim text:	
In this research, we sought to develop a data-driven and explainable computational method for understanding key factors affecting cancer trial outcomes. We applied contrast pattern mining to drug	

trials of nine individual cancer types and general cancer using four aspects of trial characteristics as feature representation of the trial documents. Our experiments and literature review demonstrated the potential of mined contrast patterns to smooth the way for identification of indicators of trial outcomes. Therefore, as an effort toward modernization of clinical trial design, our informatics framework can be further developed into a pattern recommendation system offering real-time feedback for trial investigators. Recent advances in precision oncology have revealed the increasing complexity in cancer molecular subtypes. This brings forth the emerging need of patient-centric cancer drug trials for the efficient evaluation of targeted therapies.<br><br>*Hence, the second focus of further research is to stratify cancer trials according to the molecular profiles for understanding outcome indicators specific to a certain molecular subtype. Such an effort is expected to aid in the efficient design of sub-trials aimed at identifying beneficial biomarker-drug combinations and patients who will respond to the effects of the investigational drug. The limitations of this study include the absence of factors that are not currently recorded in the trial documents, such as catchment issues for patient recruitment including geo-locations, accessibility of patients, socioeconomical status of targeting populations, sufficient number of qualified patients with driving distance to available trial sites, etc.*	
(Optional) References to other mentioned tools or studies of interest	N/A
**Section 4: Additional notes**	
Additional notes	Note on (1) Importance (re: Zarin et al): tool helps to avoid duplication, making a trial hypothesis relevant.

Section 1: Publication details	
Author(s) or Organization(s)	Clark et al.
Title of publication	Five questions that need answering when considering the design of clinical trials
Link to publication	https://doi.org/10.1186/1745-6215-15-286
Year of publication	2014
Geographic setting of the research (or, geographic affiliations of publishing authors)	Europe (Germany); UK (London)
Funder and/or sponsor of the research	Not stated
**Section 2: Intervention details**	
Type of intervention or proposed intervention to improve trial informativeness	Health Research Authority (HRA) published guidance ('Specific questions that need answering when considering the design of clinical trials')
Role of the intervention or proposed intervention within the trials research pathway	Trial design (emphasis on study protocol)
If stated, domain of study per the authors (E.g., research waste, misconduct, feasibility...)	Critical appraisal of trial design(s)
Most relevant to Zarin et al? Or, other...	All five of Zarin et al's conditions for informativeness are discussed; overview of pre-existing HRA published guidance
If applicable, population (or target population for the intervention)	Researchers, sponsors, peer reviewers and ethics committees
If applicable, method(s) used to develop the intervention	N/A; overview of pre-existing HRA published guidance
If applicable, sample size used to develop the intervention	N/A; overview of pre-existing HRA published guidance
If applicable, further details on the design or function of the proposed or actualized intervention	Five key questions within the HRA guidance:<br>1. Is there a clear research question?<br>2. Will the proposed study design answer the research question?<br>3. Are the assumptions used in the sample size calculation appropriate?<br>4. How will safety and efficacy be monitored during the trial?<br>5. How will the trial be registered and subsequently published?
If applicable, details of the evaluative process for the proposed or actualized intervention	N/A
**Section 3: Outcomes**	
If applicable, outcomes measured (or anticipated outcomes)	N/A
Author's conclusion as verbatim text:	
It is axiomatic that bad science is bad ethics. Poor research design puts us all at risk. Participants may be exposed to an inferior treatment, or enrolled in trials that provide no useful information on which to build health care. Present and future patients may receive ineffective treatment. Our collaboration provided evidence that we need to address the design of clinical trials; our guidance will help those designing such research and those reviewing it to address key issues, facilitating ethical research that will underpin and improve health care, a key role for HRA.	
(Optional) References to other mentioned tools or studies of interest	N/A
**Section 4: Additional notes**	
Additional notes	N/A

Section 1: Publication details	
Author(s) or Organization(s)	Clayton et al.
Title of publication	The INVEST project: investigating the use of evidence synthesis in the design and analysis of clinical trials
Link to publication	https://doi.org/10.1186/s13063-017-1955-y
Year of publication	2017
Geographic setting of the research (or, geographic affiliations of publishing authors)	Global [International Clinical Trials Methodology Conference (Glasgow, 2015)]
Funder and/or sponsor of the research	Medical Research Council (MRC) Hubs for Trials Methodology Research (HTMR) network
**Section 2: Intervention details**	
Type of intervention or proposed intervention to improve trial informativeness	Evidence synthesis
Role of the intervention or proposed intervention within the trials research pathway	Informing the design, conduct, and analysis of clinical trials
If stated, domain of study per the authors (E.g., research waste, misconduct, feasibility...)	Research waste
Most relevant to Zarin et al? Or, other...	(1) Importance: trial hypothesis is likely to inform an important scientific, medical, or policy decision (also see Zarin et al; (2) Design, (4) Integrity, and (5) Reporting)
If applicable, population (or target population for the intervention)	Trial teams
If applicable, method(s) used to develop the intervention	Survey (of evidence synthesis use)
If applicable, sample size used to develop the intervention	106
If applicable, further details on the design or function of the proposed or actualized intervention	INVEST (INVestigating the use of Evidence Synthesis in the design and analysis of clinical Trials) survey to summarise the current use of evidence synthesis in trial design and analysis, to capture opinions of trialists and methodologists on such use, and to understand any barriers.
If applicable, details of the evaluative process for the proposed or actualized intervention	Following details about their job role, job setting and the length of time that they had spent working in clinical trials, respondents who indicated that they had been involved in trial design (and/or analysis) were further asked questions about whether, and how, they have used evidence synthesis in practice. All respondents were then asked about their views on the use of evidence synthesis in trial design and analysis. They were also asked to rank what they considered to be the three greatest barriers to such use. There were nine potential barriers listed including an 'other' category allowing free text. The subsets of respondents who indicated that they had been involved in trial design (and/or analysis) were used to contrast views on whether evidence synthesis methods should be used versus current use in practice.
**Section 3: Outcomes**	
If applicable, outcomes measured (or anticipated outcomes)	Support was generally high for using a description of previous evidence, a systematic review or a meta-analysis in trial design. Generally, respondents did

	not seem to be using evidence syntheses as often as they felt they should. For example, only 50% (42/84 relevant respondents) had used a meta-analysis to inform whether a trial is needed compared with 74% (62/84) indicating that this is desirable. Only 6% (5/81 relevant respondents) had used a value of information analysis to inform sample size calculations versus 22% (18/81) indicating support for this. Surprisingly large numbers of participants indicated support for, and previous use of, evidence syntheses in trial analysis. For example, 79% (79/100) of respondents indicated that external information about the treatment effect should be used to inform aspects of the analysis.
Author's conclusion as verbatim text:	
Trial teams responding to the INVEST survey generally reported that they are using evidence synthesis in trial design and analysis more than we might have expected, but less than they might like to. Time constraints was identified as the greatest barrier to more widespread use. Further research on ways to undertake evidence synthesis more efficiently, and training on how to incorporate results from these into existing procedures will help to ensure the best use of relevant external evidence in the design, conduct and analysis of clinical trials.	
(Optional) References to other mentioned tools or studies of interest	N/A
**Section 4: Additional notes**	
Additional notes	N/A

Section 1: Publication details	
Author(s) or Organization(s)	De Pretto-Lazarova et al.
Title of publication	Defining clinical trial quality from the perspective of resource-limited settings: A qualitative study based on interviews with investigators, sponsors, and monitors conducting clinical trials in sub-Saharan Africa
Link to publication	https://doi.org/10.1371/journal.pntd.0010121
Year of publication	2022
Geographic setting of the research (or, geographic affiliations of publishing authors)	27 countries in sub-Saharan Africa
Funder and/or sponsor of the research	European and Developing Countries Clinical Trials Partnership (ADP), the Swiss Tropical and Public Health Institute (ADP), the Rudolf Geigy Stiftung (CB), the Freiwillige Akademische Gesellschaft (ADP), and the Stiftung Emilia Guggenheim-Schnurr (ADP)
**Section 2: Intervention details**	
Type of intervention or proposed intervention to improve trial informativeness	Comprehensive Quality Management (CQM) concept resulting from a comprehensive definition of clinical trial quality (resource-limited countries); integration to pre-existing frameworks and revisions to international guidelines
Role of the intervention or proposed intervention within the trials research pathway	CQM could be considered in the ongoing revision (R3) of the ICH-GCP E6 guideline and/or the current draft of the ICH-E8 (R1) guideline and discussed as a basis for the identification of "Critical to Quality Factors" enabling a true risk-based approach to clinical trial planning, implementation and oversight; further integration to pre-existing frameworks with a focus on resource-limited settings
If stated, domain of study per the authors (E.g., research waste, misconduct, feasibility...)	Research waste (generally); Feasibility (resource-limited settings)
Most relevant to Zarin et al? Or, other...	All five of Zarin et al's conditions for informativeness are discussed; hybrid presentation of results addressing both international guideline development and trial design considerations for resource-limited settings
If applicable, population (or target population for the intervention)	Trialists, sponsors, and monitors in sub-Saharan Africa
If applicable, method(s) used to develop the intervention	The study followed a qualitative research approach based on interviews with stakeholders having clinical trial experience in at least one country in sub-Saharan Africa.
If applicable, sample size used to develop the intervention	46 interviews (21 investigators, 13 sponsors, and 12 monitors)
If applicable, further details on the design or function of the proposed or actualized intervention	The analysis of these stakeholders' definitions of clinical trial quality has produced a Clinical Trial Quality Concept that includes quality promoting factors (i.e., Context adaptation; Infrastructure; Partnership; Operational excellence; Quality system) in addition to conventional scientific and ethical factors. [The authors] recommend the term "Comprehensive Quality Management (CQM)" for this concept.

If applicable, details of the evaluative process for the proposed or actualized intervention	N/A
**Section 3: Outcomes**	
If applicable, outcomes measured (or anticipated outcomes)	CQM has the potential to serve as a basis for the current revision of quality management principles in international clinical trial guidelines. Furthermore, the sub-Saharan African perspective has highlighted additional considerations compared to the existing comprehensive INQUIRE clinical trial quality framework. Therefore, we propose including the following three points relevant to resource-limited settings in the framework: 1) Communicating potential infrastructural disadvantages to funders, sponsors, and auditors. 2) Preventing potential exploitation of research populations and workforce in low- and middle-income countries by following existing ethical frameworks. 3) Including "Context adaptation" as an additional framework category (i.e., promoting factor).
Author's conclusion as verbatim text:	
We found that in order to enable comprehensive clinical trial quality management, clinical trial quality should be defined by a multidimensional concept that includes not only scientific and ethical, but also quality-promoting factors. Such a concept is of general relevance and not limited to clinical trials in resource-limited settings, where it naturally carries particular weight. In addition, from the perspective of sub-Saharan Africa, we identified specific categories that appear to be critical for the conduct of clinical trials in resource-limited settings, and we propose respective changes to a particular existing clinical trial quality framework (i.e., INQUIRE).	
(Optional) References to other mentioned tools or studies of interest	INQUIRE framework
**Section 4: Additional notes**	
Additional notes	N/A

Section 1: Publication details	
Author(s) or Organization(s)	Dolley et al.
Title of publication	A maturity model for the scientific review of clinical trial designs and their informativeness
Link to publication	https://doi.org/10.1186/s13063-024-08099-5
Year of publication	2024
Geographic setting of the research (or, geographic affiliations of publishing authors)	USA
Funder and/or sponsor of the research	The Bill & Melinda Gates Foundation
**Section 2: Intervention details**	
Type of intervention or proposed intervention to improve trial informativeness	Maturity model
Role of the intervention or proposed intervention within the trials research pathway	Post-funding scientific design review (prior to the start of a clinical trial)
If stated, domain of study per the authors (E.g., research waste, misconduct, feasibility…)	Informativeness
Most relevant to Zarin et al? Or, other…	All five of Zarin et al's conditions for informativeness are discussed
If applicable, population (or target population for the intervention)	Focus on funders; wider applicability to trialists and trial teams
If applicable, method(s) used to develop the intervention	To create a usable maturity model, users must carefully select the range of capacity and efforts—the cluster of related activities: in order to evaluate a scientific design review practice, the process areas must be identified and organized. At The Bill & Melinda Gates Foundation, after developing a post-funding scientific design review program across multiple disease areas and with multiple study types, eleven process areas (Pas) were identified as independent capabilities key to the program. These PAs were curated by the authors after program progress through maturity levels, participation in all areas of the program, and non-systematic interviews with other program staff. In each "cell," or capability cluster at a particular level of maturity, the contents include examples of mastery at that level. This comprehensive set offers a new or existing practitioner the benefit of including what matters and excluding what does not, resulting in time and cost savings, better clinical trials, and risk reduction. Once a maturity model variant is selected and the topic-specific PAs are populated, users can plot the maturity levels for each PA.
If applicable, sample size used to develop the intervention	N/A
If applicable, further details on the design or function of the proposed or actualized intervention	This maturity model includes 11 process areas and 5 maturity levels. Each of the 55 process area levels is populated with descriptions on a continuum toward an optimal state to improve trial protocols in the areas of risk of failure or uninformativeness.
If applicable, details of the evaluative process for the proposed or actualized intervention	N/A
**Section 3: Outcomes**	
If applicable, outcomes measured (or anticipated outcomes)	Peer-review of CTs today offers too little time for a rigorous evaluation of CT design and associated

	methods. Creating persistent improvement in a CT protocol is most likely achieved by implementing a scientific design review, and the best time for this is late in the design phase or close to when the protocol is finalized.
Author's conclusion as verbatim text:	
This tool allows for prescriptive guidance on next investments to improve attributes of post-funding reviews of trials, with a focus on informativeness. Traditional pre-funding peer review has limited capacity for trial design review, especially for detailed biostatistical and methodological review. Select non-industry funders have begun to explore or invest in post-funding review programs of grantee protocols, based on exemplars of such programs. Funders with a desire to meet fiduciary responsibilities and mission goals can use the described model to enhance efforts supporting trial participant commitment and faster cures.	
(Optional) References to other mentioned tools or studies of interest	N/A
**Section 4: Additional notes**	
Additional notes	N/A

Section 1: Publication details	
Author(s) or Organization(s)	Duley et al.
Title of publication	What are the main inefficiencies in trial conduct: a survey of UKCRC registered clinical trials units in the UK
Link to publication	https://doi.org/10.1186/s13063-017-2378-5
Year of publication	2018
Geographic setting of the research (or, geographic affiliations of publishing authors)	UK
Funder and/or sponsor of the research	No specific funding was available for this survey
**Section 2: Intervention details**	
Type of intervention or proposed intervention to improve trial informativeness	Efficient and sustainable clinical trails research as supported by Clinical Trials Units (CTUs)/networks
Role of the intervention or proposed intervention within the trials research pathway	From the beginning of a trial design process through further trial conduct and reporting
If stated, domain of study per the authors (E.g., research waste, misconduct, feasibility...)	Inefficiency in trial conduct
Most relevant to Zarin et al? Or, other...	Focus on (3) Feasibility as relevant to the rapid review, e.g., top inefficiency from recruitment of first participant to publication of results was failure to meet recruitment targets, reported by 19 (44%) respondents.
If applicable, population (or target population for the intervention)	CTUs
If applicable, method(s) used to develop the intervention	Survey
If applicable, sample size used to develop the intervention	43 respondents from 25 CTUs
If applicable, further details on the design or function of the proposed or actualized intervention	The aim was to identify important inefficiencies during two key stages of the trial conduct life cycle: (i) from grant award to first participant, (ii) from first participant to reporting of final results.
If applicable, details of the evaluative process for the proposed or actualized intervention	N/A
**Section 3: Outcomes**	
If applicable, outcomes measured (or anticipated outcomes)	
Author's conclusion as verbatim text:	
Recommendations for improving the efficiency of trial conduct within the CTUs network include: further reducing unnecessary bureaucracy in approvals and contracting; improving training for site staff; realistic recruitment targets and appropriate feasibility; developing training across the network; improving the working relationships between chief investigators and units; encouraging funders to release sufficient funding to allow prompt recruitment of trial staff; and encouraging more research into how to improve the efficiency and quality of trial conduct.	
(Optional) References to other mentioned tools or studies of interest	N/A
**Section 4: Additional notes**	
Additional notes	N/A

Section 1: Publication details	
Author(s) or Organization(s)	Erber et al.
Title of publication	Setting up a pragmatic clinical trial in a low-resource setting: A qualitative assessment of GoLBeT, a trial of podoconiosis management in Northern Ethiopia
Link to publication	https://doi.org/10.1371%2Fjournal.pntd.0009582
Year of publication	2021
Geographic setting of the research (or, geographic affiliations of publishing authors)	Ethiopia
Funder and/or sponsor of the research	Kemri Wellcome Programme
**Section 2: Intervention details**	
Type of intervention or proposed intervention to improve trial informativeness	Methodological examination of trial processes (from inception to recruitment) in a setting where research is not common
Role of the intervention or proposed intervention within the trials research pathway	Trial design; consideration of pragmatic trials in low-resource settings
If stated, domain of study per the authors (E.g., research waste, misconduct, feasibility…)	Feasibility
Most relevant to Zarin et al? Or, other…	(2) Design: trial methods are likely to provide meaningful evidence related to the study hypothesis, and equally (3) Feasibility.
If applicable, population (or target population for the intervention)	Trial teams and other public health stakeholders of local scale and scope in low-resource settings
If applicable, method(s) used to develop the intervention	Comparison with The Global Health Research Process Map (The Global Health Network); used as a comparison framework against which the real issues that occurred in GoLBeT could be compared and considered.
If applicable, sample size used to develop the intervention	Approximately 750 emails and 25 study coordinator reports relating to GoLBeT; the Principal Investigator, the Trial Coordinator and the Trial Data Manager as key members of the trial team were further consulted using interviews or an open-ended questionnaire, and a mini-group discussion was also held with three key members of the trial team.
If applicable, further details on the design or function of the proposed or actualized intervention	N/A
If applicable, details of the evaluative process for the proposed or actualized intervention	Overall differences between the process of setting up GoLBeT and the Global Health Research Process Map, see: Figure 2
**Section 3: Outcomes**	
If applicable, outcomes measured (or anticipated outcomes)	See: Table 1 (Summary of recommendations)
Author's conclusion as verbatim text:	
Lessons learnt from this trial might guide others planning pragmatic trials in settings where research is not common, allowing them to anticipate possible challenges and address them through trial design, planning and operational delivery. We also hope that this example might encourage similar pragmatic studies to be undertaken. Such studies are rarely undertaken or locally led, but are an accessible and efficient way to drive improved outcomes in public health.	
(Optional) References to other mentioned tools or studies of interest	The Global Health Research Process Map (The Global Health Network)
**Section 4: Additional notes**	
Additional notes	N/A

Section 1: Publication details	
Author(s) or Organization(s)	Heath et al.
Title of publication	Value of Information for Clinical Trial Design: The Importance of Considering All Relevant Comparators.
Link to publication	https://doi.org/10.1007/s40273-024-01372-0
Year of publication	2024
Geographic setting of the research (or, geographic affiliations of publishing authors)	UK; Canada
Funder and/or sponsor of the research	Natural Sciences and Engineering Research Council of Canada
**Section 2: Intervention details**	
Type of intervention or proposed intervention to improve trial informativeness	Value of Information (VOI) analyses
Role of the intervention or proposed intervention within the trials research pathway	VOI has been suggested as a tool for research prioritisation and trial design
If stated, domain of study per the authors (E.g., research waste, misconduct, feasibility...)	Economically valuable avenues for future research
Most relevant to Zarin et al? Or, other...	(1) Importance: trial hypothesis is likely to inform an important scientific, medical, or policy decision, and equally (2) Design: trial methods are likely to provide meaningful evidence related to the study hypothesis
If applicable, population (or target population for the intervention)	Individuals who design trials
If applicable, method(s) used to develop the intervention	All VOI analyses are based on a decision model, which aims to determine an optimal intervention from a set of alternatives (See: Section (2) Value of Information Analysis).
If applicable, sample size used to develop the intervention	N/A
If applicable, further details on the design or function of the proposed or actualized intervention	VOI offers a principled method for research prioritisation and trial design based on the net economic benefit of reducing decision uncertainty.
If applicable, details of the evaluative process for the proposed or actualized intervention	See: Section (4) Trial Analysis with a Value of Information-Based Design
**Section 3: Outcomes**	
If applicable, outcomes measured (or anticipated outcomes)	VOI methods are only accurate when (1) all feasible comparators are included in the decision model when designing research, and (2) all comparators are retained in the decision model once the data have been collected and a final treatment recommendation is made. Omitting comparators from either the design or analysis phase of research when using VOI methods can lead to incorrect trial designs and/or treatment recommendations.
Author's conclusion as verbatim text:	
VOI offers a principled method for research prioritisation and trial design based on the net economic benefit of reducing decision uncertainty. As VOI measures aim to reduce decision uncertainty, rather than demonstrate clinical effectiveness, they can provide alternative study designs to traditional methods. VOI can ensure that cost-effective interventions reach patients faster by guaranteeing that research supports policy making within publicly funded healthcare systems. However, incorrect specification of a health economic model, ignoring potential interventions or incorrectly characterising uncertainty, can lead to misleading VOI results and waste research resources. Additionally, if the trial is not analysed within the wider evidence base of the original health economic model, then ineffective interventions could be widely implemented.	

VOI analyses can lead to alternative research designs compared with standard methods that take a clinical perspective. However, they also ensure that policy decisions are formally considered in the conceptualisation and design of clinical research. This has huge potential to increase the relevance of research beyond the clinical question under consideration.	
(Optional) References to other mentioned tools or studies of interest	N/A
**Section 4: Additional notes**	
Additional notes	N/A

Section 1: Publication details	
Author(s) or Organization(s)	Heath et al.
Title of publication	Prioritisation and design of clinical trials
Link to publication	https://doi.org/10.1007%2Fs10654-021-00761-5
Year of publication	2021
Geographic setting of the research (or, geographic affiliations of publishing authors)	Europe (Netherlands); USA
Funder and/or sponsor of the research	American Diabetes Association, the Netherlands Organization for Health Research and Development, and the German Innovation Fund; Canadian Institutes of Health Research; Gordon and Betty Moore Foundation
**Section 2: Intervention details**	
Type of intervention or proposed intervention to improve trial informativeness	Two methods for the allocation of research resources (value-driven approach); review of framework for prioritisation and design of clinical trials
Role of the intervention or proposed intervention within the trials research pathway	Allocation of research resources; trial design
If stated, domain of study per the authors (E.g., research waste, misconduct, feasibility…)	Research waste
Most relevant to Zarin et al? Or, other…	(2) Design: trial methods are likely to provide meaningful evidence related to the study hypothesis (also see Zarin et al: (1) Importance, and (3) Feasibility)
If applicable, population (or target population for the intervention)	Clinical and policy decision makers
If applicable, method(s) used to develop the intervention	The value of research can be calculated using two key concepts: an estimate of the value of healthcare interventions and a suite of methods known as Value of Information (VOI) methods. See: Figure 2/Table 2 for details of the value-driven approach to prioritise and design future clinical trials.
If applicable, sample size used to develop the intervention	N/A
If applicable, further details on the design or function of the proposed or actualized intervention	See: Figure 2/Table 2
If applicable, details of the evaluative process for the proposed or actualized intervention	See: Box 2 for an example of the value-driven approach in trial design
**Section 3: Outcomes**	
If applicable, outcomes measured (or anticipated outcomes)	See: Box 2 for an example of the value-driven approach in trial design
Author's conclusion as verbatim text:	
N/A – no concluding statement provided (see below: Abstract)<br><br>*Clinical trials require participation of numerous patients, enormous research resources and substantial public funding. Time-consuming trials lead to delayed implementation of beneficial interventions and to reduced benefit to patients. This manuscript discusses two methods for the allocation of research resources and reviews a framework for prioritisation and design of clinical trials. The traditional error-driven approach of clinical trial design controls for type I and II errors. However, controlling for those statistical errors has limited relevance to policy makers. Therefore, this error-driven approach can be inefficient, waste research resources and lead to research with limited impact on daily practice. The novel value-driven approach assesses the currently available evidence and focuses on designing clinical trials that directly inform policy and treatment decisions. Estimating the net value of collecting further information, prior to undertaking a trial, informs a decision maker*	

whether a clinical or health policy decision can be made with current information or if collection of extra evidence is justified. Additionally, estimating the net value of new information guides study design, data collection choices, and sample size estimation. The value-driven approach ensures the efficient use of research resources, reduces unnecessary burden to trial participants, and accelerates implementation of beneficial healthcare interventions.	
(Optional) References to other mentioned tools or studies of interest	N/A
**Section 4: Additional notes**	
Additional notes	N/A

**Section 1: Publication details**	
Author(s) or Organization(s)	Jansen-van der Weide et al.
Title of publication	Rare disease registries: potential applications towards impact on development of new drug treatments
Link to publication	https://doi.org/10.1186/s13023-018-0836-0
Year of publication	2018
Geographic setting of the research (or, geographic affiliations of publishing authors)	Europe (Netherlands, Spain)
Funder and/or sponsor of the research	EU FP7 program
**Section 2: Intervention details**	
Type of intervention or proposed intervention to improve trial informativeness	Evaluation of existing rare disease registries (RDRs) as relevant to trial design
Role of the intervention or proposed intervention within the trials research pathway	Trial design (improving the efficiency and quality of clinical trial designs by informing the sample size calculation and expected disease course)
If stated, domain of study per the authors (E.g., research waste, misconduct, feasibility...)	Evidence synthesis
Most relevant to Zarin et al? Or, other...	(2) Design: trial methods are likely to provide meaningful evidence related to the study hypothesis, and equally (5) Reporting: systems are in place to ensure timely, complete and accurate reporting
If applicable, population (or target population for the intervention)	Researchers, regulators, and funders
If applicable, method(s) used to develop the intervention	Focus groups; interviews; use of European Public Assessment Reports describing examples in which RDR data had been used for drug approval
If applicable, sample size used to develop the intervention	Two focus groups with 3 different statisticians each and an interview with two regulatory experts with extensive experience at the European Medicines agency
If applicable, further details on the design or function of the proposed or actualized intervention	Low prevalence, lack of knowledge about the disease course, and phenotype heterogeneity hamper the development of drugs for rare diseases. Rare disease registries can be helpful by playing a role in understanding the course of the disease, and providing information necessary for clinical trial design, if designed and maintained properly.
If applicable, details of the evaluative process for the proposed or actualized intervention	This overview was developed by experts from the Asterix consortium. Asterix is an EU funded consortium that focuses on the development of research methodologies for rare disease drug trials. The Asterix team comprises statisticians, methodologists, patient representatives, regulators, and clinicians, all with expertise in the field of rare diseases.<br><br>Focus groups and interviews were first conducted to investigate the RDR applications and to develop a checklist of elements to record. Possible relevant information from models from the literature was added to the checklist, which was discussed by e-mail until there was consensus. Finally, the completeness of two existing RDRs was checked against trial design.

**Section 3: Outcomes**	
If applicable, outcomes measured (or anticipated outcomes)	Before and during the application for regulatory approval a RDR can improve the efficiency and quality in clinical trial design by informing the sample size calculation and expected disease course. In exceptional circumstances information from RDRs has been used as historical controls for a one-armed clinical trial, and high quality RDRs may be used for registry-based randomized controlled trials. In the post marketing phase of (conditional) drug approval a disease-specific RDR is likely to provide more relevant information than a product-specific registry.
Author's conclusion as verbatim text:	
A RDR can be very helpful in trial design by informing the sample size calculation, it can increase efficiency by being a data collection tool in clinical trials, may provide a historical control group in instances when placebo or active comparators are e.g. not ethically acceptable, and it can be informative in the post marketing phase.<br><br>*To enable the applicability and optimal use of a RDR longitudinal data collection is indispensable, and specific data collection, prepared for repeated measurement, is needed. The developed checklist can help to define the appropriate variables to include.*<br>*Disease-specific RDRs are preferred over product-specific registries. In a disease-specific RDR all consenting patients with the disease are included, and not only the patients who receive a certain treatment.*<br><br>*Valid measurement instruments should be used, and measurements, data collection and data management should make use of global data standards to optimise comparability with clinical trial data.*	
(Optional) References to other mentioned tools or studies of interest	N/A
**Section 4: Additional notes**	
Additional notes	N/A

**Section 1: Publication details**	
Author(s) or Organization(s)	Jull et al.
Title of publication	When is a randomised controlled trial health equity relevant? Development and validation of a conceptual framework
Link to publication	https://doi.org/10.1136/bmjopen-2016-015815
Year of publication	2017
Geographic setting of the research (or, geographic affiliations of publishing authors)	Global
Funder and/or sponsor of the research	Not stated
**Section 2: Intervention details**	
Type of intervention or proposed intervention to improve trial informativeness	Health equity framework (conceptual)
Role of the intervention or proposed intervention within the trials research pathway	Trial design and reporting
If stated, domain of study per the authors (E.g., research waste, misconduct, feasibility...)	Health equity
Most relevant to Zarin et al? Or, other...	(2) Design: trial methods are likely to provide meaningful evidence related to the study hypothesis, and equally (5) Reporting: systems are in place to ensure timely, complete and accurate reporting
If applicable, population (or target population for the intervention)	Trialists, funders, journal editors and other knowledge user groups
If applicable, method(s) used to develop the intervention	An interdisciplinary and international research team engaged in an iterative consensus building process to develop and refine the conceptual framework via face-to-face meetings, teleconferences and email correspondence, including findings from a validation exercise whereby two independent reviewers used the emerging framework to classify a sample of randomised trials.
If applicable, sample size used to develop the intervention	N/A – no further sample size details provided on the research team engaged in framework development.
If applicable, further details on the design or function of the proposed or actualized intervention	The proposed conceptual framework engages users in a deliberative process to design and report studies to provide evidence that is useful to understand the distribution of effects rather than only average effects.
If applicable, details of the evaluative process for the proposed or actualized intervention	Validation exercise undertaken by two independent reviewers using the conceptual framework to classify a sample of randomised trials; limitations noted include the analysis and publishing of disaggregated data can be expensive and may not be desirable for all randomised trials.
**Section 3: Outcomes**	
If applicable, outcomes measured (or anticipated outcomes)	The conceptual framework may be used to design health equity-relevant randomised trials (as well as other study types) and to identify health equity-relevant studies that contribute to an evidence base that improves overall health and health equity.
Author's conclusion as verbatim text:	
The conceptual framework defines features of health equity-relevant trials that can be used to design and report both randomised trials and other study designs to improve the evidence base about how to improve health equity. Opportunities to provide health equity-relevant evidence should be considered upfront in trial concept and design. The conceptual framework can provide a stimulus to build	

knowledge about effects of interventions on health equity and is a first step to improve application of effective interventions and to build evidence that is of greatest relevance and use to individuals and populations.	
(Optional) References to other mentioned tools or studies of interest	PROGRESS-Plus
**Section 4: Additional notes**	
Additional notes	N/A

Section 1: Publication details	
Author(s) or Organization(s)	Kavalci & Hartshorn
Title of publication	Improving clinical trial design using interpretable machine learning based prediction of early trial termination
Link to publication	https://doi.org/10.1038/s41598-023-27416-7
Year of publication	2023
Geographic setting of the research (or, geographic affiliations of publishing authors)	UK (London, England)
Funder and/or sponsor of the research	Not stated
**Section 2: Intervention details**	
Type of intervention or proposed intervention to improve trial informativeness	Machine learning
Role of the intervention or proposed intervention within the trials research pathway	Outset of a trial design process (prepare for potential recruitment issues, suggesting direct changes to the study design if possible)
If stated, domain of study per the authors (E.g., research waste, misconduct, feasibility...)	Research waste
Most relevant to Zarin et al? Or, other...	(2) Design: trial methods are likely to provide meaningful evidence related to the study hypothesis, and (3) Feasibility: the trial is likely to be feasible
If applicable, population (or target population for the intervention)	Clinical researchers
If applicable, method(s) used to develop the intervention	The goal is to predict early termination probability of clinical trials using machine learning modelling, and to understand feature contributions driving early termination. This will inform further suggestions to the study protocol to reduce the risk of wasted resources. A dataset containing 420,268 clinical trial records and 24 fields was extracted from the ct.gov registry. In addition to study characteristics features, 12,864 eligibility criteria search features are used, generated using a public annotated eligibility criteria dataset, CHIA. Furthermore, disease categorization features are used allowing a study to belong more than one category specified by clinicaltrials.gov. Ensemble models including random forest and extreme gradient boosting classifiers were used to train and evaluate predictive performance. We achieved a Receiver Operator Characteristic Area under the Curve score of 0.80, and balanced accuracy of 0.70 on the test set using gradient boosting classification. We used Shapley Additive Explanations to interpret the termination predictions to flag feature contributions.
If applicable, sample size used to develop the intervention	420,268 clinical trials from the AACT database (ClinicalTrials.gov)
If applicable, further details on the design or function of the proposed or actualized intervention	Proposed 3-stage clinical trial optimisation pipeline: 1) data preparation and analytics; 2) machine learning pipeline and 3) interpret predictions. See: Figure 1.
If applicable, details of the evaluative process for the proposed or actualized intervention	McNemar's Test; machine learning classification results; phase specific datasets generated and models trained and evaluated; a comparison of the

	state-of-the-art models; interpreting predictions with Shapley Additive Explanations
**Section 3: Outcomes**	
If applicable, outcomes measured (or anticipated outcomes)	See: Author's conclusion
Author's conclusion as verbatim text:	
In this study, we extracted 420,268 clinical trials from the AACT database and used feature engineering methods on numerical, categorical and free-text columns, and used machine learning to predict early trial termination using these features. We proposed adding eligibility criteria search features generated from free text columns and disease categorization features to the study characteristics features and showed that this approach is statistically significant to increase the early clinical trial termination prediction performance. We achieved 80% ROC-AUC, 70% balanced accuracy and 42% as the F1 score on the xgBoost model trained using all phases dataset. Finally, we used SHAP explanations to transform our black-box machine learning models into insightful suggestions towards the clinical study protocols. Eligibility criteria and design features are flagged as contributions towards success or failure during the interpretation process. These flagged features might help prepare for potential recruitment issues, as well as suggesting direct changes to the study design if possible. Hence, this pipeline provides an optimised machine learning based trial design process.	
(Optional) References to other mentioned tools or studies of interest	N/A
**Section 4: Additional notes**	
Additional notes	N/A

Section 1: Publication details	
Author(s) or Organization(s)	Khalil et al.
Title of publication	The role of scoping reviews in reducing research waste
Link to publication	https://doi.org/10.1016/j.jclinepi.2022.09.012
Year of publication	2022
Geographic setting of the research (or, geographic affiliations of publishing authors)	Global
Funder and/or sponsor of the research	Tier 2 Canada Research Chair in Knowledge Synthesis; NHMRC investigator grant
**Section 2: Intervention details**	
Type of intervention or proposed intervention to improve trial informativeness	Scoping review(s)
Role of the intervention or proposed intervention within the trials research pathway	Trial design
If stated, domain of study per the authors (E.g., research waste, misconduct, feasibility...)	Research waste
Most relevant to Zarin et al? Or, other...	(1) Importance: trial hypothesis is likely to inform an important scientific, medical, or policy decision
If applicable, population (or target population for the intervention)	N/A; summary of issues regarding research waste and how scoping reviews can make an importance contribute to reduction of research waste in both primary and secondary research
If applicable, method(s) used to develop the intervention	N/A; see above
If applicable, sample size used to develop the intervention	N/A; see above
If applicable, further details on the design or function of the proposed or actualized intervention	N/A; see above
If applicable, details of the evaluative process for the proposed or actualized intervention	N/A; see above
**Section 3: Outcomes**	
If applicable, outcomes measured (or anticipated outcomes)	N/A; see above
Author's conclusion as verbatim text:	
The problem of research waste is an enduring challenge for global health, leading to a waste of human and financial resources and producing research outputs that do not provide answers to the most pressing research questions. Research waste occurs within primary research but also in secondary research such as evidence syntheses. The focus of scoping reviews on characterizing the nature of existing evidence on a topic and including all types of evidence, potentially reduces research waste in five ways: (1) identifying key research gaps on a topic, (2) determining appropriate outcome measures, (3) mapping existing methodological approaches, (4) developing a consistent understanding of terms and concepts used in existing evidence, and (5) ensuring scoping reviews do not exacerbate the issue of research waste. To ensure that scoping reviews do not themselves end up contributing to research waste, it is important to register the scoping review and to ensure that international reporting standards and methodological guidance are followed.	
(Optional) References to other mentioned tools or studies of interest	N/A
**Section 4: Additional notes**	
Additional notes	N/A

Section 1: Publication details	
Author(s) or Organization(s)	Lane et al.
Title of publication	Approaches for enhancing the informativeness and quality of clinical trials: Innovations and principles for implementing multicenter trials from the Trial Innovation Network
Link to publication	https://doi.org/10.1017%2Fcts.2023.560
Year of publication	2023
Geographic setting of the research (or, geographic affiliations of publishing authors)	USA
Funder and/or sponsor of the research	National Institutes of Health Center for Advancing Translational Sciences (NCATS) and the National Institute on Aging (NIA)
**Section 2: Intervention details**	
Type of intervention or proposed intervention to improve trial informativeness	Approaches for enhancing the informativeness of clinical trials
Role of the intervention or proposed intervention within the trials research pathway	Focus on ensuring the conditions of an informative trial are incorporated into all aspects of trial planning and execution
If stated, domain of study per the authors (E.g., research waste, misconduct, feasibility...)	Informativeness
Most relevant to Zarin et al? Or, other...	All five of Zarin et al's conditions of informativeness are broadly discussed, in addition to more practical considerations including funding.
If applicable, population (or target population for the intervention)	Investigators who are proposing multicenter collaborations
If applicable, method(s) used to develop the intervention	Draws on the experience of the National Center for Advancing Translational Science (NCATS) Trial Innovation Network (TIN) to develop approaches for enhancing the informativeness of clinical trials.
If applicable, sample size used to develop the intervention	TIN, comprised of NCATS, three Trial Innovation Centers, a Recruitment Innovation Center, and 60+ CTSA Program hubs
If applicable, further details on the design or function of the proposed or actualized intervention	Three key guiding principles: (1) assemble a diverse team, (2) leverage existing processes and systems, and (3) carefully consider budgets and contracts.
If applicable, details of the evaluative process for the proposed or actualized intervention	N/A
**Section 3: Outcomes**	
If applicable, outcomes measured (or anticipated outcomes)	See: Author's conclusion
Author's conclusion as verbatim text:	
Clinical trials are an important part of the translational science spectrum. Single center studies may be advantageous, especially when testing a new research hypothesis in a small population. For small-scale studies, a single center can be more homogeneous in equipoise, training, and management. A single center can also be more efficient: funding needs are smaller and can be easier to obtain, the study will be easier to conduct, and IRB approvals can be secured more quickly.<br><br>*When it becomes important to generalize findings in more diverse populations and settings, larger multicenter trials come into play. Multicenter trials have many benefits, such as a larger number of participants, different geographic locations, inclusion of a wider range of population groups, and the ability to compare results among centers, which will increase the overall generalizability and informativeness of the trial results. However, multicenter trials require more resources and more diverse training and management than single center studies. Inefficient and ineffective processes in multicenter trials can lead to uninformative results. When setting up a multicenter trial, early*	

considerations include determining the timelines and costs for developing the critical processes associated with receiving and analyzing study data, providing logistical support to physically distant colleagues and personnel at multiple institutions, and documenting operating procedures to ensure a systematic approach to all trial procedures. Diverse teams with complementary expertise leveraging existing processes and systems can shorten timelines and hold down costs.<br><br>*The NIH recognizes the importance of multicenter trial planning time and factors this into the structure of awards for multicenter trials. Planning time for multicenter trials is needed so that investigative teams can engage collaborators, obtain regulatory approvals, and train center staff. The TIN recognizes that many multicenter investigative teams may not know how to efficiently and effectively plan for all the challenges associated with multicenter studies. The TIN has therefore developed many resources to help investigative teams learn how to track their planning period progress, prioritize tasks, and use reasonable time estimates when setting timelines and milestones.*<br><br>*The TIN has published extensively about its work, including barriers and roadblocks in clinical trial operations. Many TIN resources are publicly available via the online TIN Toolbox, and training events are also advertised on the TIN website, with previous events archived for ongoing access. Trial investigators seeking a formal TIN consultation can collaborate with their local CTSA that will review and provide local resources or support a submission to the TIN portal. CTSA liaison teams are listed on the TIN website to help investigators connect with local resources and support.*<br><br>*The breadth and complexity of multicenter studies can stress resources and will challenge the skillsets of even very experienced multicenter trialists. To have the best chance of conducting an informative multicenter trial, every PI, regardless of experience, must assemble early a diverse team and empower the team to plan and to confer with colleagues, funding officers, and CTSA staff to jointly define solutions before there are problems, and to develop a realistic budget that matches the details and needs of the trial. The CTSA Program hubs and the TIN are resources that can help investigative teams initiate multicenter trials, the cornerstone of informative, evidence-based medicine.*	
(Optional) References to other mentioned tools or studies of interest	N/A
**Section 4: Additional notes**	
Additional notes	N/A

Section 1: Publication details	
Author(s) or Organization(s)	Leiter et al.
Title of publication	Use of Crowdsourcing for Cancer Clinical Trial Development
Link to publication	https://doi.org/10.1093/jnci/dju258
Year of publication	2014
Geographic setting of the research (or, geographic affiliations of publishing authors)	USA
Funder and/or sponsor of the research	Prostate Cancer Foundation Young Investigator Award
**Section 2: Intervention details**	
Type of intervention or proposed intervention to improve trial informativeness	Crowdsourcing platform
Role of the intervention or proposed intervention within the trials research pathway	Informing trial design from inception; focus on protocol development and amendments
If stated, domain of study per the authors (E.g., research waste, misconduct, feasibility…)	Protocol design
Most relevant to Zarin et al? Or, other…	All five of Zarin et al's conditions of informativeness are broadly discussed with an emphasis on (1) Importance.
If applicable, population (or target population for the intervention)	Physicians, researcher, patients, survivors, and advocates
If applicable, method(s) used to develop the intervention	A secure web-based platform (Transparency Life Sciences, New York, NY) that enabled participants to provide input (through closed- and open-ended responses) regarding important design elements of a planned clinical trial.
If applicable, sample size used to develop the intervention	60 physicians/researchers; 42 patients/advocates
If applicable, further details on the design or function of the proposed or actualized intervention	Input on key elements of a trial's design is typically provided by a small group of researchers. Patient feedback is usually limited to the few patient advocates involved in academic or industry protocol review committees. Crowdsourcing allows for the completion of a task by obtaining input from a large number of individuals.
If applicable, details of the evaluative process for the proposed or actualized intervention	Tested in real-time on a planned clinical trial exploring metformin in prostate cancer. Members of the Mount Sinai Genitourinary Oncology Research Team wrote a complete study protocol prior to initiation of the crowdsourcing effort.
**Section 3: Outcomes**	
If applicable, outcomes measured (or anticipated outcomes)	Crowdsourcing input led to nine total changes (four major and five minor) to the original protocol, including modifications to eligibility criteria and study procedures. Agreement among three independent reviewers scoring the number of protocol modifications was excellent (eight, nine, and nine changes). Notably, 91% of physicians/researchers and 76% of patients/advocates agreed or strongly agreed with the statement, "I would participate in a similar clinical trial crowdsourcing effort in the future."
Author's conclusion as verbatim text:	

Crowdsourcing clinical trial design is feasible, adds value to the protocol development process, and may ultimately improve the efficiency of trial conduct.	
(Optional) References to other mentioned tools or studies of interest	N/A
**Section 4: Additional notes**	
Additional notes	N/A

Section 1: Publication details	
Author(s) or Organization(s)	Li et al.
Title of publication	Incorporating ethical principles into clinical research protocols: a tool for protocol writers and ethics committees
Link to publication	https://jme.bmj.com/content/42/4/229
Year of publication	2016
Geographic setting of the research (or, geographic affiliations of publishing authors)	USA
Funder and/or sponsor of the research	Not commissioned
**Section 2: Intervention details**	
Type of intervention or proposed intervention to improve trial informativeness	Protocol Ethics Tool Kit ('Ethics Tool Kit')
Role of the intervention or proposed intervention within the trials research pathway	To facilitate effective recognition, consideration and deliberation of critical ethical issues in the development of clinical trial protocols
If stated, domain of study per the authors (E.g., research waste, misconduct, feasibility...)	Ethics
Most relevant to Zarin et al? Or, other...	(2) Design: trial methods are likely to provide meaningful evidence related to the study hypothesis, and equally, (4) Integrity: trial is conducted and analyzed in a scientifically valid manner that is faithful to design
If applicable, population (or target population for the intervention)	Individual protocol writers and study teams, study sponsors, and ethics committees.
If applicable, method(s) used to develop the intervention	Developed by a multi-stakeholder group of the Multi-Regional Clinical Trials Center of Brigham and Women's Hospital and Harvard; (1) formation of a working group, (2) literature review and (3) review of a sample of 100 approved clinical trial protocols. Following these initial steps, the multi-stakeholder working group aggregated, aligned and reviewed focused ethical questions that were then formatted as (4) an Ethics Tool Kit and accompanying guidance document to allow dynamic usage by protocol writers and ethics committees alike.
If applicable, sample size used to develop the intervention	A group of 20 experts from academic institutions (6), pharmaceutical companies (4), non-profit organisations (4), law firms (3) and ethics committees (3), with backgrounds in clinical trials, medicine, bioethics and law was formed by the MRCT Center in 2012 to create a list of ethical elements that should be addressed when writing and/or reviewing a clinical trial protocol. Each member introduced potential ethical elements by drawing upon the research ethics literature and existing sponsor protocol templates, areas identified as confusing by ethics committees and domestic and international guidelines. Initially, all recommendations from all 20 members were compiled. The ethics elements were then discussed, challenged and categorised to appropriately group similar elements and reduce redundancy. The working group met a total of 24 times over a period of approximately 18 months by teleconference. One in-person meeting was also held

	to reach consensus on issues that could not be reconciled earlier. The 'Essential Elements' that comprise the basis of the Ethics Tool Kit were then compiled, annotated, reviewed and refined.
If applicable, further details on the design or function of the proposed or actualized intervention	The Ethics Tool Kit is structured in such a way that it can be adapted to meet an individual user's needs and address specific challenges. Each Essential Element has (1) a short explanation, (2) specific points to consider, (3) background information, (4) practical examples and (5) references. See: Tables 2 and 3.
If applicable, details of the evaluative process for the proposed or actualized intervention	A total of 100 clinical trial protocols were reviewed to determine if the 11 Essential Elements the working group drafted were present in the current approved clinical trial protocols and, if they were, whether they were discussed explicitly from an ethical perspective.
**Section 3: Outcomes**	
If applicable, outcomes measured (or anticipated outcomes)	See Table 1.
Author's conclusion as verbatim text:	
• Substantive discussion of specific ethical issues is rarely included in clinical trial protocols.<br>• A total of 11 'Essential Elements' have been identified that should be considered and addressed as appropriate in a clinical trial protocol.<br>• The Protocol Ethics Tool Kit has been developed to support protocol writers, study teams, sponsors, ethics committees and reviewers.<br>• Use of this tool could result in more efficient development and review of clinical trial protocols and may result in wider appreciation of the ethical challenges in clinical research.	
(Optional) References to other mentioned tools or studies of interest	N/A
**Section 4: Additional notes**	
Additional notes	Link to the Ethics Tool Kit appears defunct; http://mrctcenter.org/resources/2014-11-14-training-material-mrct-ethics-essential-elements-and-points-to-consider-reference-document-toolkit/

**Section 1: Publication details**	
Author(s) or Organization(s)	Loudon et al.
Title of publication	The PRECIS-2 tool: designing trials that are fit for purpose
Link to publication	https://doi.org/10.1136/bmj.h2147
Year of publication	2015
Geographic setting of the research (or, geographic affiliations of publishing authors)	UK (Scotland) and Canada
Funder and/or sponsor of the research	Chief Scientist Office (CSO) of Scotland, UK Medical Research Council, and the University of Dundee work through the provision of a PhD stipend for KL and from the Health Services Research Unit at the University of Aberdeen, which is core funded by the CSO of the Scottish Government Health Directories
**Section 2: Intervention details**	
Type of intervention or proposed intervention to improve trial informativeness	Trial design tool
Role of the intervention or proposed intervention within the trials research pathway	To be used at the design stage of a trial in order to help trialists make the purpose of their trial explicit and to ensure that their design choices are concordant with their intended purpose.
If stated, domain of study per the authors (E.g., research waste, misconduct, feasibility...)	Research waste
Most relevant to Zarin et al? Or, other...	(2) Design: trial methods are likely to provide meaningful evidence related to the study hypothesis (see also Zarin et al; (3) Feasibility, and (4) Integrity)
If applicable, population (or target population for the intervention)	Trialists
If applicable, method(s) used to develop the intervention	Delphi study, user testing
If applicable, sample size used to develop the intervention	19 (user testing)
If applicable, further details on the design or function of the proposed or actualized intervention	Four step, iterative process to using PRECIS-2 utilizing nine domains intended to help trialists think about the consequences of that design decision for applicability of the results of their trial. The PRECIS-2 tool is focused exclusively on the issue of applicability, and other tools should be used to assess the internal validity or any other aspect of design choices.
If applicable, details of the evaluative process for the proposed or actualized intervention	See: Examples provided across all nine domains.
**Section 3: Outcomes**	
If applicable, outcomes measured (or anticipated outcomes)	PRECIS-2 has nine domains—eligibility criteria, recruitment, setting, organisation, flexibility (delivery), flexibility (adherence), follow-up, primary outcome, and primary analysis—scored from 1 (very explanatory) to 5 (very pragmatic) to facilitate domain discussion and consensus.
Author’s conclusion as verbatim text:	
PRECIS-2 has been developed through extensive international consultation with trialists, and we believe it will help trialists (new and experienced) ensure that they match their design decisions to the needs of those they intend to use the results of the trial. It makes trial teams explicitly aware of the range of opinions within the team and facilitates discussion and eventual consensus. The tool aims to assist in obtaining consistency in decision making, meaning decisions made on each domain of	

PRECIS-2 should broadly be in keeping with each other. It does not remove the need for judgment because there is no single "correct" answer to be discovered. The advantage of PRECIS-2 is that it makes these judgments explicit and therefore able to be discussed by the trial team.<br><br>*PRECIS-2 could be a tool in a multifaceted package to help trialists design trials that meet the users' needs. For instance, in the US, the NIH Clinical and Translational Science Awards (CTSA) Consortium does not conduct trials but facilitates their conduct and implementation, and PRECIS-2 could used as part of those activities. Trials units and other centres of trial expertise could work through the PRECIS-2 wheel with investigators to ensure that trial design decisions are consistent with the investigators' intended purpose. The tool could also be a way of promoting trials that efficiently assist in the continued development of evidence based care. It would thus continue to promote and re-energise Archie Cochrane's vision of efficient effective healthcare by addressing the issue of applicability and extrapolating trial results into real life.*<br><br>*There is a substantial degree of waste in medical research (see http://researchwaste.net). Some of this waste is due to trials providing results that are irrelevant to the healthcare decisions of those for whom the trial was intended to help. We believe PRECIS-2 can help to reduce this waste by raising awareness of the need to explicitly consider the match between design decisions (and the consequences of these) and the usefulness of the future results to the intended audience. As Dave Sackett has pointed out, the applicability of the results of a trial can never be assured because of the complexity of patients, health professionals, clinical settings, cultures, and healthcare systems. But using PRECIS-2 to consider applicability at the design stage will make it more likely that the results of a trial are more useful to their intended users than they otherwise would have been.*	
(Optional) References to other mentioned tools or studies of interest	N/A
**Section 4: Additional notes**	
Additional notes	Note: ST is an author on this paper.

**Section 1: Publication details**	
Author(s) or Organization(s)	McFadden et al.
Title of publication	The impact of registration of clinical trials units: The UK experience
Link to publication	https://doi.org/10.1177/1740774514561242
Year of publication	2014
Geographic setting of the research (or, geographic affiliations of publishing authors)	UK
Funder and/or sponsor of the research	UK Clinical Research Collaboration (UKCRC)–Registered Clinical Trials Unit (CTU) Network
**Section 2: Intervention details**	
Type of intervention or proposed intervention to improve trial informativeness	Clinical research networks (registration programme)
Role of the intervention or proposed intervention within the trials research pathway	Registration programme for Clinical Trials Units involved in the coordination of clinical trials
If stated, domain of study per the authors (E.g., research waste, misconduct, feasibility...)	Compliance (registration process developed in the United Kingdom has helped to ensure that trials units in the United Kingdom are compliant with regulatory standards and can meet acceptable standards of quality in their conduct of clinical trial)
Most relevant to Zarin et al? Or, other...	(4) Integrity: trial is conducted and analyzed in a scientifically valid manner that is faithful to the design *(Integrity of *trial units*)
If applicable, population (or target population for the intervention)	Clinical trial units
If applicable, method(s) used to develop the intervention	Applications for registration were invited from all active, non-commercial Clinical Trials Units in the United Kingdom. The invitations were issued in 2007, 2009 and 2012, and applicants were asked to describe their expertise and staffing levels in specific areas. To ensure that the reviews were as objective as possible, a description of expected core competencies was developed and applicants were asked to document their compliance with meeting these. The review panel assessed each Clinical Trials Unit against the competencies
If applicable, sample size used to develop the intervention	2007 – 48 applications<br>2009 – 31 applications<br>2012 – 55 applications
If applicable, further details on the design or function of the proposed or actualized intervention	See Table 1: Core competencies and evaluation criteria (2012)
If applicable, details of the evaluative process for the proposed or actualized intervention	See Table 1: Core competencies and evaluation criteria (2012)
**Section 3: Outcomes**	
If applicable, outcomes measured (or anticipated outcomes)	See Table 1: Core competencies and evaluation criteria (2012)
Author's conclusion as verbatim text:	
The registration process developed in the United Kingdom has helped to ensure that trials units in the United Kingdom are compliant with regulatory standards and can meet acceptable standards of quality in their conduct of clinical trials. There is an increased awareness among funders, host institutions and Clinical Trials Units themselves of the required competencies, and communication between all those involved in trials has increased. The registration process is an effective and financially viable way of ensuring that objective standards are met at a national level.	
(Optional) References to other mentioned tools or studies of interest	N/A

Section 4: Additional notes	
Additional notes	N/A

**Section 1: Publication details**	
Author(s) or Organization(s)	Meeker-O'Connell et al.
Title of publication	Enhancing clinical evidence by proactively building quality into clinical trials
Link to publication	https://doi.org/10.1177%2F1740774516643491
Year of publication	2016
Geographic setting of the research (or, geographic affiliations of publishing authors)	USA
Funder and/or sponsor of the research	United States Food and Drug Administration
**Section 2: Intervention details**	
Type of intervention or proposed intervention to improve trial informativeness	Quality-by-design principles
Role of the intervention or proposed intervention within the trials research pathway	Protocol design, trial planning, and quality oversight
If stated, domain of study per the authors (E.g., research waste, misconduct, feasibility...)	Quality management
Most relevant to Zarin et al? Or, other...	All five conditions of informativeness as described by Zarin et al. are broadly discussed.
If applicable, population (or target population for the intervention)	Stakeholders across the clinical trial enterprise
If applicable, method(s) used to develop the intervention	A working group evaluated aspects of trial design and oversight and developed the Clinical Trials Transformation Initiative quality-by-design principles document, outlining a series of factors generally relevant to the reliability of trial conclusions and to patient safety.
If applicable, sample size used to develop the intervention	The CTTI quality-by-design principles were developed collaboratively by industry, regulators, and academia and were refined during a series of hands-on workshops involving over 200 attendees with a range of perspectives (including patient advocates, institutional review boards, academic trialists, clinical investigators, clinical research organizations, pharmaceutical and medical device companies, regulatory reviewers, and inspectors). Small, cross-disciplinary groups applied the principles to mock clinical trial protocol synopses covering various development phases, disease conditions product types. Independent qualitative interviews were conducted with 19 attendees to explore the challenges of implementing a quality-by-design approach to clinical trials within academic and commercial organizations.
If applicable, further details on the design or function of the proposed or actualized intervention	Key principles were then applied and further refined during a series of hands-on workshops to evaluate their utility in facilitating proactive, cross-functional dialogue, and decision-making about trial design and planning.
If applicable, details of the evaluative process for the proposed or actualized intervention	Following these workshops, independent qualitative interviews were conducted with 19 workshop attendees to explore the potential challenges for implementing a quality-by-design approach to clinical trials. The Clinical Trials Transformation Initiative project team subsequently developed

	recommendations and an online resource guide to support implementation of this approach.
**Section 3: Outcomes**	
If applicable, outcomes measured (or anticipated outcomes)	See Figure 1 & Figure 2:<br>1) Create a culture that values and rewards critical thinking and open dialogue about quality, and that goes beyond sole reliance on tools and checklists.<br>2) Focus effort on activities that are essential tot the credibility of the study outcomes.<br>3) Involve the broad range of stakeholders in protocol development and discussions around study quality.<br>4) Prospectively identify and periodically review factors critical to quality.
Author's conclusion as verbatim text:	
The Clinical Trials Transformation Initiative quality-by-design principles provide a framework for assuring that clinical trials adequately safeguard participants and provide reliable information on which to make decisions on the effects of treatments. The quality-by-design workshops highlighted the value of active discussions incorporating the different perspectives within and external to an organization (e.g. clinical investigators, research site staff, and trial participants) in improving trial design. Workshop participants also recognized the value of focusing oversight on those aspects of the trial where errors would have a major impact on participant safety and reliability of results. Applying the Clinical Trials Transformation Initiative quality-by-design recommendations and principles should enable organizations to prioritize the most critical determinants of a trial's quality, identify non-essential activities that can be eliminated to streamline trial conduct and oversight, and formulate appropriate plans to define, avoid, mitigate, monitor, and address important errors.	
(Optional) References to other mentioned tools or studies of interest	N/A
**Section 4: Additional notes**	
Additional notes	N/A

Section 1: Publication details	
Author(s) or Organization(s)	Morgan et al.
Title of publication	Do feasibility studies contribute to, or avoid, waste in research?
Link to publication	https://doi.org/10.1371/journal.pone.0195951
Year of publication	2018
Geographic setting of the research (or, geographic affiliations of publishing authors)	UK
Funder and/or sponsor of the research	Not stated
**Section 2: Intervention details**	
Type of intervention or proposed intervention to improve trial informativeness	Feasibility study
Role of the intervention or proposed intervention within the trials research pathway	Feasibility study before a clinical trial
If stated, domain of study per the authors (E.g., research waste, misconduct, feasibility...)	Research waste
Most relevant to Zarin et al? Or, other...	(3) Feasibility: the trial is likely to be feasible
If applicable, population (or target population for the intervention)	Principle investigators
If applicable, method(s) used to develop the intervention	Feasibility studies funded by the National Institute for Health Research's (NIHR) Research for Patient Benefit (RfPB) programme were examined to determine how many had published their findings, how many had applied for further funding for a full trial and the timeframe in which both of these occurred.
If applicable, sample size used to develop the intervention	A total of 120 feasibility studies which had closed by May 2016 were identified and each Principal Investigator (PI) was sent a questionnaire of which 89 responses were received and deemed suitable for analysis.
If applicable, further details on the design or function of the proposed or actualized intervention	See: Tables 1-5
If applicable, details of the evaluative process for the proposed or actualized intervention	See: Discussion, Abstract (below)
**Section 3: Outcomes**	
If applicable, outcomes measured (or anticipated outcomes)	See: Discussion, Abstract (below)
Author's conclusion as verbatim text:	

N/A – no concluding statement provided (see below: Abstract)

In the context of avoiding research waste, the conduct of a feasibility study before a clinical trial should reduce the risk that further resources will be committed to a trial that is likely to 'fail'. However, there is little evidence indicating whether feasibility studies add to or reduce waste in research. Feasibility studies funded by the National Institute for Health Research's (NIHR) Research for Patient Benefit (RfPB) programme were examined to determine how many had published their findings, how many had applied for further funding for a full trial and the timeframe in which both of these occurred. A total of 120 feasibility studies which had closed by May 2016 were identified and each Principal Investigator (PI) was sent a questionnaire of which 89 responses were received and deemed suitable for analysis. Based on self reported answers from the PIs a total of 57 feasibility studies were judged as feasible, 20 were judged not feasible and for 12 it was judged as uncertain whether a full trial was feasible. The RfPB programme had spent approximately £19.5m on the 89 feasibility studies of which 16 further studies had been subsequently funded to a total of £16.8m. The 20 feasibility studies which were judged as not feasible potentially saved up to approximately £20m of further research funding which would likely to

have not completed successfully. The average RfPB feasibility study took 31 months (range 18 to 48) to complete and cost £219,048 (range £72,031 to £326,830) and the average full trial funded from an RfPB feasibility study took 42 months (range 26 to 55) to complete and cost £1,163,996 (range £321,403 to £2,099,813). The average timeframe of feasibility study and full trial was 72 months (range 56 to 91), however in addition to this time an average of 10 months (range -7 to 29) was taken between the end of the feasibility study and the application for the full trial, and a further average of 18 months (range 13 to 28) between the application for the full trial and the start of the full trial. Approximately 58% of the 89 feasibility studies had published their findings with the majority of the remaining studies still planning to publish. Due to the long time frames involved a number of studies were still in the process of publishing the feasibility findings and/or applying for a full trial. Feasibility studies are potentially useful at avoiding waste and de-risking funding investments of more expensive full trials, however there is a clear time delay and therefore some potential waste in the existing research pathway.	
(Optional) References to other mentioned tools or studies of interest	CONSORT
**Section 4: Additional notes**	
Additional notes	N/A

**Section 1: Publication details**	
Author(s) or Organization(s)	Natafgi et al.
Title of publication	Patients' early engagement in research proposal development (PEER-PD): patients guiding the proposal writing
Link to publication	https://doi.org/10.2217/cer-2018-0129
Year of publication	2019
Geographic setting of the research (or, geographic affiliations of publishing authors)	USA
Funder and/or sponsor of the research	Agency for Healthcare Research and Quality; Patient-Centered Outcomes Research Institute
**Section 2: Intervention details**	
Type of intervention or proposed intervention to improve trial informativeness	Patients' early engagement in research proposal development process and its contemporary relevance to clinical and translational research
Role of the intervention or proposed intervention within the trials research pathway	Research proposal development
If stated, domain of study per the authors (E.g., research waste, misconduct, feasibility...)	Patient engagement
Most relevant to Zarin et al? Or, other...	(1) Importance: trial hypothesis is likely to inform an important scientific, medical, or policy decision
If applicable, population (or target population for the intervention)	Patients and investigators
If applicable, method(s) used to develop the intervention	N/A; comparison of different levels of engagement and description of the effect of patients' early engagement in research proposal development (PEER-PD) on the development of a research proposal and its contemporary relevance to clinical and translational research.
If applicable, sample size used to develop the intervention	N/A; see above
If applicable, further details on the design or function of the proposed or actualized intervention	N/A; see above
If applicable, details of the evaluative process for the proposed or actualized intervention	N/A; see above
**Section 3: Outcomes**	
If applicable, outcomes measured (or anticipated outcomes)	See Table 1: Examples of patient engagement along a 10-step research continuum
Author's conclusion as verbatim text:	
It is well to remember the words of Robert Moses: *"I have no fear of change as such and, on the other hand, no liking for it merely for its own sake"*. As humans, we both fear and anticipate change. That dichotomy applies to the introduction of patient engagement to the research process. Although there are challenges to PEER-PD, none is insurmountable and the benefits of adding the patient voice to the development of concepts and protocols outweigh the challenges. In the patient's own words: "*Now, I recognize and appreciate that research studies and their results aren't just abstract good and useful things; rather, in significant ways they define and extend my life, enriching my ability to be a contributing member of society and part of a rich community network. Sometimes, it's a medicine I take; other times, it's a lifestyle change I've made because a carefully constructed study has given me new, life-enhancing options"*.	
(Optional) References to other mentioned tools or studies of interest	N/A
**Section 4: Additional notes**	
Additional notes	N/A

**Section 1: Publication details**	
Author(s) or Organization(s)	Parks Taylor & Kowalkowski
Title of publication	Using Implementation Science-Guided Pilot Studies to Assess and Improve the Informativeness of Clinical Trials
Link to publication	https://doi.org/10.1007/s11606-020-06220-3
Year of publication	2020
Geographic setting of the research (or, geographic affiliations of publishing authors)	USA
Funder and/or sponsor of the research	Not stated
**Section 2: Intervention details**	
Type of intervention or proposed intervention to improve trial informativeness	Implementation science-guided pilot studies
Role of the intervention or proposed intervention within the trials research pathway	Early in study development (pilot study)
If stated, domain of study per the authors (E.g., research waste, misconduct, feasibility...)	Informativeness
Most relevant to Zarin et al? Or, other...	All five of Zarin et al's conditions for informativeness are broadly discussed.
If applicable, population (or target population for the intervention)	Researchers planning RCTs (of complex interventions)
If applicable, method(s) used to develop the intervention	N/A; proposed use of implementation science to inform pre-RCT pilot studies
If applicable, sample size used to develop the intervention	N/A; proposed use of implementation science to inform pre-RCT pilot studies
If applicable, further details on the design or function of the proposed or actualized intervention	Implementation science-guided pilot studies should consider the following:<br>1) Measure and report implementation outcomes<br>2) Apply a conceptual framework<br>3) Use the pilot study implementation findings to inform and adapt the planned RCT<br>4) Report pilot study results including qualitative and quantitative findings
If applicable, details of the evaluative process for the proposed or actualized intervention	See above
**Section 3: Outcomes**	
If applicable, outcomes measured (or anticipated outcomes)	See above
Author's conclusion as verbatim text:	
Uninformative clinical trials are a major challenge in medicine. Carefully designed, conducted, and interpreted pilot studies can be important foundations for successful clinical trials. Using implementation science to guide these pre-RCT pilot studies may improve the value of investments in RCTs by addressing contextual factors influencing the informativeness of RCT results to the healthcare delivery community and its patients.	
(Optional) References to other mentioned tools or studies of interest	
**Section 4: Additional notes**	
Additional notes	References Zarin et al and the concept of 'informativeness' directly within the text.

**Section 1: Publication details**	
Author(s) or Organization(s)	Pirosca et al.
Title of publication	Learning from Cochrane systematic reviews: what improvements do these suggest for the design of trials?
Link to publication	https://doi.org/10.12688/f1000research.22635.2
Year of publication	2020
Geographic setting of the research (or, geographic affiliations of publishing authors)	UK
Funder and/or sponsor of the research	Health Research Board as part of Trial Forget (non-commercial research initiative)
**Section 2: Intervention details**	
Type of intervention or proposed intervention to improve trial informativeness	Summary/relevance of *'Implications for research'* as stated in Cochrane systematic reviews
Role of the intervention or proposed intervention within the trials research pathway	Trial design (priority areas for improvement)
If stated, domain of study per the authors (E.g., research waste, misconduct, feasibility...)	Trial methodology
Most relevant to Zarin et al? Or, other...	(2) Design: trial methods are likely to provide meaningful evidence related to the study hypothesis
If applicable, population (or target population for the intervention)	Trialists and others working in the area of trials (e.g., funders)
If applicable, method(s) used to develop the intervention	Reviews with citation dates between 2009 and 2019 were identified and the recommendations of review authors in *'Implications for research'* were put into categories.
If applicable, sample size used to develop the intervention	206 Cochrane systematic reviews identified between 2009 and 2019
If applicable, further details on the design or function of the proposed or actualized intervention	22 categories of recommendations in total
If applicable, details of the evaluative process for the proposed or actualized intervention	N/A; Summary/relevance of *'Implications for research'* as stated in Cochrane systematic reviews
**Section 3: Outcomes**	
If applicable, outcomes measured (or anticipated outcomes)	The five most used categories were: better choice of outcomes; better choice of intervention/comparator; longer follow-up; larger sample size; use of validated scales. Better choice of outcomes and/or intervention/comparator was recommended in over 50% of reviews. Longer follow-up and larger sample size were recommended in over a third, with use of validated scales being suggested in around a fifth of reviews. There was no obvious pattern of improvement over time for trials included in systematic reviews.
Author's conclusion as verbatim text:	
We suggest that trialists working in these and other areas ask themselves, or are compelled to do so by others (e.g. funders), why they have chosen their outcomes, intervention and comparator, whether follow-up is long enough, if the sample size is big enough and whether the scales they choose to measure their outcomes are appropriate.	
(Optional) References to other mentioned tools or studies of interest	PRECIS-2; COMET
**Section 4: Additional notes**	
Additional notes	Note: ST is an author on this paper.

Section 1: Publication details	
Author(s) or Organization(s)	Robert et al.
Title of publication	ASCO's Community Research Forum: addressing challenges of community-based research from the grass roots
Link to publication	https://doi.org/10.14694/edbook_am.2014.34.e111
Year of publication	2014
Geographic setting of the research (or, geographic affiliations of publishing authors)	USA
Funder and/or sponsor of the research	Not stated
**Section 2: Intervention details**	
Type of intervention or proposed intervention to improve trial informativeness	Research forum
Role of the intervention or proposed intervention within the trials research pathway	Trial design and conduct
If stated, domain of study per the authors (E.g., research waste, misconduct, feasibility...)	Community research
Most relevant to Zarin et al? Or, other...	(1) Importance: trial hypothesis is likely to inform an important scientific, medical, or policy decision, (2) Design: trial methods are likely to provide meaningful evidence related to the study hypothesis, and equally, (3) Feasibility: the trial is likely to be feasible.
If applicable, population (or target population for the intervention)	Physician investigators as well as research staff (i.e., research administrators, research nurses, and clinical research associates) from any type of community-based research site and/or program that is not academic-based and is currently conducting clinical trials
If applicable, method(s) used to develop the intervention	ASCO's Community Research Forum was conceived from ASCO Board of Directors' Clinical Trials Strategic Plan of 2010, which in part defined ASCO's role in supporting clinical investigators and increasing their participation in cancer clinical trials. In response, ASCO's Cancer Research Committee convened an advisory council in December of 2010 to implement the Community Research Forum as a solution-oriented venue for community research sites to overcome barriers to conducting clinical trials.
If applicable, sample size used to develop the intervention	N/A; further needs of forum members were identified by a total of 123 surveys and resulted in the development of two tools for use in research/trial design and management and to further explore a research certification program for community-based programs.
If applicable, further details on the design or function of the proposed or actualized intervention	The three key objectives of the Forum are to (1) convene community-based researchers to identify challenges to conducting research that ASCO can address, (2) develop solution-oriented projects to address these challenges and facilitate clinical trial participation in community research settings, and (3) shape ASCO programs and policies to support members engaged in community research.
If applicable, details of the evaluative process for the proposed or actualized intervention	N/A

Section 3: Outcomes	
If applicable, outcomes measured (or anticipated outcomes)	N/A
Author's conclusion as verbatim text:	
The ASCO Community Research Forum provides a means for ASCO to fulfill its mission of supporting members at community-based practices. The Forum aims to provide community-based physician investigators and research staff with a venue to discuss their challenges around conducting clinical trials and develop solution-oriented initiatives. Tools and resources continue to be developed and made available as ASCO-sponsored products to the membership at large. For more information, visit the Community Research Forum website at www.asco.org/communityresearchforum.	
(Optional) References to other mentioned tools or studies of interest	ASCO Clinical Trial Workload Assessment Tool and the ASCO Research Program Quality Assessment Tool
**Section 4: Additional notes**	
Additional notes	N/A

Section 1: Publication details	
Author(s) or Organization(s)	Sharma et al.
Title of publication	Using HTA and guideline development as a tool for research priority setting the NICE way: reducing research waste by identifying the right research to fund
Link to publication	https://doi.org/10.1136/bmjopen-2017-019777
Year of publication	2017
Geographic setting of the research (or, geographic affiliations of publishing authors)	UK
Funder and/or sponsor of the research	This research received no specific grant from any funding agency in the public, commercial or not-for-profit sectors.
**Section 2: Intervention details**	
Type of intervention or proposed intervention to improve trial informativeness	Health Technology Assessment and guideline development as a tool for research priority setting
Role of the intervention or proposed intervention within the trials research pathway	Ensuring appropriate research priority setting occurs so only the questions that are needed to fill existing gaps in the evidence are funded.
If stated, domain of study per the authors (E.g., research waste, misconduct, feasibility...)	Research waste
Most relevant to Zarin et al? Or, other...	(1) Importance: trial hypothesis is likely to inform an important scientific, medical, or policy decision
If applicable, population (or target population for the intervention)	National Institute for Health and Care Excellence (NICE) and related funding bodies (e.g., the NIHR, NHS, MRC)
If applicable, method(s) used to develop the intervention	NICE uses its guidance production processes to identify and prioritise research questions through systematic reviews, economic analyses and stakeholder consultations and then highlights those priorities by engagement with the research community. NICE also highlights its methodological areas for research to ensure the appropriate development and growth of the evidence landscape. Methodological priorities were established by an Internal Research Advisory Group.
If applicable, sample size used to develop the intervention	N/A
If applicable, further details on the design or function of the proposed or actualized intervention	Research recommended by NICE has been systematically prioritised through evidence reviews and stakeholder input; formal prioritization processes support the 'importance' criteria of informativeness. See Figure 1 for further details on the 7-step research recommendations process.
If applicable, details of the evaluative process for the proposed or actualized intervention	NICE has prioritised research questions through its guidance production and methodological work and has successfully had several research products funded through the NIHR and MRC.
**Section 3: Outcomes**	
If applicable, outcomes measured (or anticipated outcomes)	Research priorities identified through systematic reviews; and, if appropriate, health economic modelling and cost-effectiveness decision analysis presented using GRADE profiles that allow for a transparent representation of the confidence in the evidence available for decision-making.

	Methodological priorities categorized by 9 areas: (1) real-world evidence; (2) data science; (3) adaptive pathways; (4) patient preferences; (5) improvements in cross-sector comparisons; (6) expert elicitation; (7) complex data visualisation; (8) precision medicine; and (9) implementation of NICE guidance.
Author's conclusion as verbatim text:	
It is important that HTA and guideline organisations use their systematic processes to identify research gaps and then subsequently link with national research funders to ensure they are addressed. NICE therefore supports the reducing research waste campaign by ensuring that the research it recommends has a beneficial impact on the health and care of the people, as it has been systematically identified as a genuine gap in the health evidence or a method that needs further clarification or development.	
(Optional) References to other mentioned tools or studies of interest	N/A
**Section 4: Additional notes**	
Additional notes	N/A

Section 1: Publication details	
Author(s) or Organization(s)	Suls et al.
Title of publication	Now is the time to assess the effects of open science practices with randomized control trials
Link to publication	https://doi.org/10.1037/amp0000871
Year of publication	2021
Geographic setting of the research (or, geographic affiliations of publishing authors)	USA
Funder and/or sponsor of the research	National Institute on Aging (R24AG064191) and the National Library of Medicine (R01LM012836) of the National Institutes of Health
**Section 2: Intervention details**	
Type of intervention or proposed intervention to improve trial informativeness	Open Science (OS) research practices
Role of the intervention or proposed intervention within the trials research pathway	N/A; noted by the authors as a 'call to action rather than an action plan'
If stated, domain of study per the authors (E.g., research waste, misconduct, feasibility...)	Open Science
Most relevant to Zarin et al? Or, other...	Focus on (1) Importance: trial hypothesis is likely to inform an important scientific, medical, or policy decisions.
If applicable, population (or target population for the intervention)	Scientists, funders, policymakers, and institutions
If applicable, method(s) used to develop the intervention	OS practices extend and formalize traditional research practices, partly by capitalizing on opportunities afforded by advances in technology. For example, researchers are now encouraged to preregister their prospective project on online platforms or submit a registered report, where hypotheses, experimental design, and analytic plans are specified prior to data collection. They are also encouraged to use repositories that afford access to methods and data. See Table 1 for fuller description of OS research practices.
If applicable, sample size used to develop the intervention	N/A; noted by the authors as a 'call to action rather than an action plan'
If applicable, further details on the design or function of the proposed or actualized intervention	OS represents an umbrella term for a variety of activities, policies and resources that are directed toward both individual researchers (e.g., preregistration) and organizations ranging from scientific societies and publishers to funding agencies (e.g., open access journals), signed reviews, and public data repositories.
If applicable, details of the evaluative process for the proposed or actualized intervention	Potential use of RCT format to evaluate OS practices; conceptualization of four potential trial designs to encourage further deliberation and planning
**Section 3: Outcomes**	
If applicable, outcomes measured (or anticipated outcomes)	See Figure 1 (Outcomes: Productivity Outcomes; Impact; Reproducibility Outcomes; Shareable Outcomes; Translational Outcomes; Repurposing of Data; Shifts in Research Areas).
Author's conclusion as verbatim text:	
OS represents a movement that could improve the entire scientific enterprise. The OS researcher practices, already being implemented by some scientists, represent one key component. Though researchers may not fully understand whether OS, in its fullest and most robust form, confers multiple	

benefits (or unforeseen harms), this important hypothesis merits experimental testing. The enterprise we propose is not a "one-off" project and does not involve testing a single hypothesis because there are multiple OS practices (see Table 1) and many possible short- and long-term outcomes. However, experimental meta-science can provide data about which practices confer greater benefits and fewer downsides. A larger outcome may be a consensus about practices that might be uniformly implemented, but additionally which practices should be implemented under more limited conditions. In turn, all segments of the scientific community—scientists, funders, policymakers, and institutions—can optimize engagement in and valuing of OS research practices. Taking a longer view, experimental testing of OS practices should be embedded within a wider ranging activity whereby scientific disciplines and institutions engage in an ongoing process of self-evaluation and improvement through experimentation.	
(Optional) References to other mentioned tools or studies of interest	N/A
**Section 4: Additional notes**	
Additional notes	N/A

**Section 1: Publication details**	
Author(s) or Organization(s)	Swezey et al.
Title of publication	More than a box to check: Research sponsor and clinical investigator perspectives on making GCP training relevant.
Link to publication	https://doi.org/10.1016/j.conctc.2020.100606
Year of publication	2020
Geographic setting of the research (or, geographic affiliations of publishing authors)	USA; Canada
Funder and/or sponsor of the research	Food and Drug Administration
**Section 2: Intervention details**	
Type of intervention or proposed intervention to improve trial informativeness	Good Clinical Practice (GCP) training
Role of the intervention or proposed intervention within the trials research pathway	Relevant training to ensure quality conduct of registrational clinical trials
If stated, domain of study per the authors (E.g., research waste, misconduct, feasibility...)	Quality management
Most relevant to Zarin et al? Or, other...	(4) Integrity: trial is conducted and analyzed in a scientifically valid manner that is faithful to the design *(Integrity of *individuals)*
If applicable, population (or target population for the intervention)	Clinical investigators and their delegates
If applicable, method(s) used to develop the intervention	Qualitative semi-structured interviews
If applicable, sample size used to develop the intervention	13 clinical investigators and 10 research sponsors
If applicable, further details on the design or function of the proposed or actualized intervention	Interviews intended to 1) examine characteristics of the quality conduct of sponsored clinical trials, including critical tasks and concerns perceived as essential for trial quality, 2) identify key knowledge and skills required to perform critical tasks, and 3) identify gaps and redundancies in GCP training and areas of improvement to ensure quality conduct of clinical trials.
If applicable, details of the evaluative process for the proposed or actualized intervention	Data were examined using applied thematic analysis.
**Section 3: Outcomes**	
If applicable, outcomes measured (or anticipated outcomes)	The top three tasks identified as critical for the quality conduct of clinical trials were obtaining informed consent, ensuring protocol compliance, and protecting participants' health and safety. Respondents acknowledged that GCP principles address each of these critical tasks but also described many challenges and burdens of GCP training, including high training frequency and repetitive content. Respondents suggested moving beyond GCP training as a mere check-box activity by making it more effective, engaging, and interactive. They also emphasized that applying GCP principles in a real-world, skills-based environment would increase the perceived relevance of GCP training.
Author’s conclusion as verbatim text:	
Our findings indicate that although investigators and sponsors recognize that GCP training addresses tasks critical to the quality conduct of clinical trials, the need for significant improvement in the design, content, and presentation of GCP training remains.	

(Optional) References to other mentioned tools or studies of interest	N/A
**Section 4: Additional notes**	
Additional notes	N/A

Section 1: Publication details	
Author(s) or Organization(s)	Tagaris et al.
Title of publication	PAT: An Intelligent Authoring Tool for Facilitating Clinical Trial Design
Link to publication	10.3233/978-1-61499-432-9-970
Year of publication	2014
Geographic setting of the research (or, geographic affiliations of publishing authors)	Europe (Greece)
Funder and/or sponsor of the research	European Commission's 7th Framework Programme
**Section 2: Intervention details**	
Type of intervention or proposed intervention to improve trial informativeness	PONTE Authoring Tool (PAT)
Role of the intervention or proposed intervention within the trials research pathway	Authoring of clinical trial protocols
If stated, domain of study per the authors (E.g., research waste, misconduct, feasibility...)	Facilitation of clinical trial designs (with a focus on feasibility)
Most relevant to Zarin et al? Or, other...	With the exception of (5) Reporting, all elements of informativeness as relevant to Zarin et al are discussed.
If applicable, population (or target population for the intervention)	Emphasis on collaborative authoring of protocols for multi-center trials
If applicable, method(s) used to develop the intervention	1) interviews with clinical research experts; 2) analysis of information available on ClinicalTrials.gov; 3) use of Model Veiw Controll Design pattern approach to implement the PAT as a web tool.
If applicable, sample size used to develop the intervention	1) 8 clinical research experts; 2) 1000 criteria selected across all studies on ClinicalTrials.gov
If applicable, further details on the design or function of the proposed or actualized intervention	In brief, PAT allows PIs to manage their clinical trial protocols (CTPs) and the healthcare entities they co-operate with for patient recruitment purposes. It presents the user with three different CTP views: a hierarchical one (based on the CTP structure), a dependency one (based on dependencies between parameters) and a semantic one (built around three key concepts: the investigational active substance, the study disorder and the drug target). Hence, the PIs are offered a very flexible interface to navigate across the study parameters and fill in their data. Validation checks are provided whenever PIs save their work at a section or the CTP as a whole. For each section, PAT presents to the PI a set of automatically generated questions, the answer to which, may facilitate their (research) work. These questions are directly linked with the semantic search engine GoPONTE, to automatically retrieve the results of the selected ones.
If applicable, details of the evaluative process for the proposed or actualized intervention	PAT encapsulates intelligent, semantically-assisted mechanisms for study parameter<br>specification and EC determination. The thorough analysis of CTP templates<br>performed by experienced clinical experts, allowed for the development of a model,<br>based on which PAT organizes CTPs around 3

	different views: the hierarchy of its parameters, dependencies among them and their semantic linking with the CTP main concepts (investigational active substance, study disorder and drug target). These views were considered as "very helpful" during trial design by the experts, as they allowed for "easy and fast detection of inconsistencies" and "less time consuming parameter specification" although the dependencies should be further enriched and the 3 main concepts could be expanded to also include "clinical trials" and "patient" concepts.
**Section 3: Outcomes**	
If applicable, outcomes measured (or anticipated outcomes)	The CTP parameters are directly linked with literature through templates of questions that researchers would be interested in finding answers for, during design. According to the clinical experts, this functionality boost the scientific validity of the parameters and allows for faster referencing of the CTP.
Author's conclusion as verbatim text:	
Analysis of the eligibility criteria (EC) across studies published at clinicaltrials.gov, together with reviewing of the extracted patterns by clinical experts, led to a series of EC templates for describing the study population. PAT links with mechanisms applying these criteria onto patient data at healthcare entities (i.e., hospitals, clinics, etc.) in order to provide an estimation of the eligible population size. This functionality was considered of great value by the clinical experts (especially PIs) as it provides an indication about the feasibility of the study as well as the potential market share. Furthermore, it offers automatic retrieval of eligible patients who could potentially participate in the trial. According to the clinical experts, this is expected to significantly reduce the patient recruitment resources, both in terms of time and effort required, but also, will allow for fast and effective site recruitment. The EC model and resulting templates were evaluated as well structured and capable to cover frequently used criteria with a modest degree of complexity. However, further analysis is required in order to capture even more complicated EC but also to enrich the underlying vocabularies.	
(Optional) References to other mentioned tools or studies of interest	N/A
**Section 4: Additional notes**	
Additional notes	N/A

**Section 1: Publication details**	
Author(s) or Organization(s)	Taylor et al.
Title of publication	Embedding stakeholder preferences in setting priorities for health research: Using a discrete choice experiment to develop a multi-criteria tool for evaluating research proposals
Link to publication	https://doi.org/10.1371/journal.pone.0295304
Year of publication	2023
Geographic setting of the research (or, geographic affiliations of publishing authors)	New Zealand; Australia
Funder and/or sponsor of the research	The authors received no specific funding for this work.
**Section 2: Intervention details**	
Type of intervention or proposed intervention to improve trial informativeness	Multi-criteria tool for evaluating research proposals
Role of the intervention or proposed intervention within the trials research pathway	Trial proposal development
If stated, domain of study per the authors (E.g., research waste, misconduct, feasibility…)	Research waste (prioritization of research)
Most relevant to Zarin et al? Or, other…	(1) Importance: trial hypothesis is likely to inform an important scientific, medical, or policy decision
If applicable, population (or target population for the intervention)	Clinical trial stakeholders (broadly)
If applicable, method(s) used to develop the intervention	Cross-sectional, adaptive discrete choice experiment
If applicable, sample size used to develop the intervention	220 complete survey responses
If applicable, further details on the design or function of the proposed or actualized intervention	Weighted identification from four criteria – Appropriateness, Significance, Relevance, Feasibility so that research proposals can be scored between 0% (nil or very low merit) and 100% (very high merit).
If applicable, details of the evaluative process for the proposed or actualized intervention	See above; the tool can be used to assess the relative merits of clinical trial research proposals and rank them, to help identify the best proposals for funding.
**Section 3: Outcomes**	
If applicable, outcomes measured (or anticipated outcomes)	In designing the tool, the most important criterion was *Appropriateness* (adjusted for differences between stakeholder groups, mean weight 28.9%) and the least important was *Feasibility* (adjusted mean weight 19.5%). Consumers tended to weight *Relevance* more highly (2.7% points difference) and *Feasibility* less highly (3.1% points difference) than researchers.
Author's conclusion as verbatim text:	
N/A – no concluding statement provided (see below: Abstract)<br><br>*We determined weights for a multi-criteria tool for assessing the relative merits of clinical-trial research proposals, and investigated whether the weights vary across relevant stakeholder groups. A cross-sectional, adaptive discrete choice experiment using 1000minds online software was administered to consumers, researchers and funders affiliated with the Australian Clinical Trials Alliance (ACTA). We identified weights for four criteria—Appropriateness, Significance, Relevance, Feasibility—and their levels, representing their relative importance, so that research proposals can be scored between 0% (nil or very low merit) and 100% (very high merit). From 220 complete survey responses, the most important criterion was Appropriateness (adjusted for differences between stakeholder groups, mean weight 28.9%) and the least important was Feasibility (adjusted mean weight 19.5%). Consumers*	

tended to weight Relevance more highly (2.7% points difference) and Feasibility less highly (3.1% points difference) than researchers. The research or grant writing experience of researchers or consumers was not associated with the weights. A multi-criteria tool for evaluating research proposals that reflects stakeholders' preferences was created. The tool can be used to assess the relative merits of clinical trial research proposals and rank them, to help identify the best proposals for funding.	
(Optional) References to other mentioned tools or studies of interest	
**Section 4: Additional notes**	
Additional notes	

Section 1: Publication details	
Author(s) or Organization(s)	Totton et al.
Title of publication	Appropriate design and reporting of superiority, equivalence and non-inferiority clinical trials incorporating a benefit risk assessment: the BRAINS study including expert workshop
Link to publication	https://doi.org/10.3310/bhqz7691
Year of publication	2023
Geographic setting of the research (or, geographic affiliations of publishing authors)	UK
Funder and/or sponsor of the research	MRC–NIHR Methodology Research Programme
**Section 2: Intervention details**	
Type of intervention or proposed intervention to improve trial informativeness	Benefit-risk methods
Role of the intervention or proposed intervention within the trials research pathway	Aid the design of clinical trials with multiple outcomes of interest by defining when each trial design is appropriate to use and identifying when to use benefit-risk methods to assess outcome trade-offs (qualitatively or quantitatively) in a publicly funded trial setting.
If stated, domain of study per the authors (E.g., research waste, misconduct, feasibility…)	Trial design
Most relevant to Zarin et al? Or, other…	(2) Design: trial methods are likely to provide meaningful evidence related to the study hypothesis, and equally, (4) Integrity: trial is conducted and analyzed in a scientifically valid manner that is faithful to design (also see Zarin et al; (5) Reporting).
If applicable, population (or target population for the intervention)	Relevant trials researchers and experts
If applicable, method(s) used to develop the intervention	A range of methods was used to elicit expert opinion to answer the project objectives, including a web-based survey of relevant researchers, a rapid review of current literature and a 2-day consensus workshop of experts (in 2019).
If applicable, sample size used to develop the intervention	Web-based survey (n= 64); 2-day expert consensus workshop (n = 15)
If applicable, further details on the design or function of the proposed or actualized intervention	A list of 19 factors to aid researchers in selecting the most appropriate trail design was created containing the following overarching sections: population, intervention, comparator, outcomes, feasibility and perspectives.
If applicable, details of the evaluative process for the proposed or actualized intervention	Six key reasons that indicate a benefit-risk method should be considered within a trial were identified: (1) when the success of the trial depends on more than one outcome; (2) when important outcomes within the trial are in competing directions (i.e. a health technology is better for one outcome, but worse for another); (3) to allow patient preferences to be included and directly influence trial results; (4) to provide transparency on subjective recommendations from a trial; (5) to provide consistency in the approach to presenting results from a trial; and (6) to synthesise multiple outcomes into a single metric. Further information was provided to support the use of benefit-risk methods in

	appropriate circumstances, including the following: methods identified from the review were collated into different groupings and described to aid the selection of a method; potential implementation of methods throughout the trial process were provided and discussed (with examples); and general considerations were described for those using benefit-risk methods. Finally, a checklist of five pieces of information that should be present when reporting benefit-risk methods was defined, with two additional items specifically for reporting the results.
**Section 3: Outcomes**	
If applicable, outcomes measured (or anticipated outcomes)	See above.
Author's conclusion as verbatim text:	
These recommendations will assist research teams in selecting which trial design to use and deciding whether or not a benefit-risk method could be included to ensure research questions are answered appropriately. Additional information is provided to support consistent use and clear reporting of benefit-risk methods in the future. The recommendations can also be used by funding committees to confirm that appropriate considerations of the trial design have been made.	
(Optional) References to other mentioned tools or studies of interest	N/A
**Section 4: Additional notes**	
Additional notes	N/A

**Section 1: Publication details**	
Author(s) or Organization(s)	Toye et al.
Title of publication	What Value Can Qualitative Research Add to Quantitative Research Design? An Example From an Adolescent Idiopathic Scoliosis Trial Feasibility Study
Link to publication	https://doi.org/10.1177/1049732316662446
Year of publication	2016
Geographic setting of the research (or, geographic affiliations of publishing authors)	UK
Funder and/or sponsor of the research	NIHR
**Section 2: Intervention details**	
Type of intervention or proposed intervention to improve trial informativeness	Qualitative research
Role of the intervention or proposed intervention within the trials research pathway	Inclusion of qualitative research in trial design
If stated, domain of study per the authors (E.g., research waste, misconduct, feasibility...)	Trial design
Most relevant to Zarin et al? Or, other...	(2) Design: trial methods are likely to provide meaningful evidence related to the study hypothesis
If applicable, population (or target population for the intervention)	Trial stakeholders
If applicable, method(s) used to develop the intervention	Interviews (*note: uses an example of qualitative research embedded in a non-surgical feasibility trial*)
If applicable, sample size used to develop the intervention	18 trial participants
If applicable, further details on the design or function of the proposed or actualized intervention	To make the most out of qualitative research embedded in quantitative design it would be useful to (a) agree specific qualitative study aims that underpin research design, (b) understand the impact of differences in epistemological truth claims, (c) provide clear thematic interpretations for trial researchers to utilize, and (d) include qualitative findings that explore experience beyond the trial setting within the impact plan.
If applicable, details of the evaluative process for the proposed or actualized intervention	Interpretive Phenomenological Analysis
**Section 3: Outcomes**	
If applicable, outcomes measured (or anticipated outcomes)	Findings demonstrated that qualitative research can make a valuable contribution by allowing trial stakeholders to see things from alternative perspectives. Specifically, it can help to make specific recommendations for improved trial design, generate questions which contextualize findings, and also explore disease experience beyond the trial.
Author's conclusion as verbatim text:	
N/A – no concluding statement provided (see below: Abstract)<br><br>*Using an example of qualitative research embedded in a non-surgical feasibility trial, we explore the benefits of including qualitative research in trial design and reflect on epistemological challenges. We interviewed 18 trial participants and used methods of Interpretive Phenomenological Analysis. Our findings demonstrate that qualitative research can make a valuable contribution by allowing trial stakeholders to see things from alternative perspectives. Specifically, it can help to make specific recommendations for improved trial design, generate questions which contextualize findings, and also explore disease experience beyond the trial. To make the most out of qualitative research embedded in quantitative design it would be useful to (a) agree specific qualitative study aims that underpin research*	

design, (b) understand the impact of differences in epistemological truth claims, (c) provide clear thematic interpretations for trial researchers to utilize, and (d) include qualitative findings that explore experience beyond the trial setting within the impact plan.	
(Optional) References to other mentioned tools or studies of interest	N/A
**Section 4: Additional notes**	
Additional notes	N/A

Section 1: Publication details	
Author(s) or Organization(s)	van Iersel et al.
Title of publication	The Patient Motivation Pyramid and Patient-Centricity in Early Clinical Development
Link to publication	10.2174/1574884716666210427115820
Year of publication	2022
Geographic setting of the research (or, geographic affiliations of publishing authors)	USA; Europe (Netherlands, Belgium)
Funder and/or sponsor of the research	ICON plc (USA)
**Section 2: Intervention details**	
Type of intervention or proposed intervention to improve trial informativeness	Patient Motivation Pyramid based on Maslow's theory of human motivation
Role of the intervention or proposed intervention within the trials research pathway	This pyramid is used to make a comprehensive overview of options to implement a patient-centric trial design in early phase development.
If stated, domain of study per the authors (E.g., research waste, misconduct, feasibility...)	Feasibility
Most relevant to Zarin et al? Or, other...	(2) Design: trial methods are likely to provide meaningful evidence related to the study hypothesis, and equally, (3) Feasibility: the trial is likely to be feasible
If applicable, population (or target population for the intervention)	Trialists
If applicable, method(s) used to develop the intervention	Based on Maslow's theory of human motivation; specific methods are not further described by the authors.
If applicable, sample size used to develop the intervention	N/A
If applicable, further details on the design or function of the proposed or actualized intervention	Pyramid is comprised of five differing patients' needs based on Maslow's Theory of human motivation; 1) convenience, 2) risk/benefit, 3) social interaction, 4) partnership, 5) altruism. For each level of the pyramid the authors propose specific recommendations as to how a patient-centric approach can be integrated to trial design. For example, in the first level of the pyramid (convenience) decreasing the burden for patients through options such as decentralized trials and providing practical support including financial incentives.
If applicable, details of the evaluative process for the proposed or actualized intervention	Three case examples are presented; the first example is seeking patient input on the trial design for a First-in-Human trial which includes patients with Immune Thrombocytopenic Purpura. The second example is the use of a video-link for home dosing. The final example is the use of digital medicine in a decentralized trial in heart failure patients.
**Section 3: Outcomes**	
If applicable, outcomes measured (or anticipated outcomes)	The authors note that patient input can lead to improved endpoints, improved feasibility, better recruitment, less dropout, less protocol amendments, and higher patient satisfaction. Outcomes would vary by indication.
Author's conclusion as verbatim text:	
Satisfying the needs of the end-user (the patient) should be the focus of drug development. In all stages of clinical development, a patient-centric trial design is an obvious choice since patients have the	

experience of living with the condition and are able to provide the true picture of what it is like to live with a specific condition and how that impacts them, their family and other caregivers. A comprehensive overview of patients' needs can be accomplished by building a Patient Motivation Pyramid based on Maslow's theory of human motivation as a tool. We recommend this structural approach for identifying elements of patient-centricity relevant to the specific drug development. Secondly, we recommend starting using patient-centric approaches already in an early phase of the medicine's lifecycle.	
(Optional) References to other mentioned tools or studies of interest	N/A
**Section 4: Additional notes**	
Additional notes	N/A

**Section 1: Publication details**	
Author(s) or Organization(s)	Vischer et al.
Title of publication	Increasing protocol suitability for clinical trials in sub-Saharan Africa: a mixed methods study
Link to publication	https://doi.org/10.1186/s41256-017-0031-1
Year of publication	2017
Geographic setting of the research (or, geographic affiliations of publishing authors)	sub-Saharan Africa
Funder and/or sponsor of the research	R. Geigy Foundation; Burckhardt-Bürgin-Stiftung; Freiwillige Akademische Gesellschaft
**Section 2: Intervention details**	
Type of intervention or proposed intervention to improve trial informativeness	Measures to enhance trial protocol suitability
Role of the intervention or proposed intervention within the trials research pathway	Protocol development
If stated, domain of study per the authors (E.g., research waste, misconduct, feasibility...)	Quality through suitability of trial design (re: Sub-Saharan Africa [SSA]).
Most relevant to Zarin et al? Or, other...	(2) Design: trial methods are likely to provide meaningful evidence related to the study hypothesis, and equally, (3) Feasibility: the trial is likely to be feasible.
If applicable, population (or target population for the intervention)	Trial teams
If applicable, method(s) used to develop the intervention	Exploratory mixed methods design; interviews and online (quantitative) survey
If applicable, sample size used to develop the intervention	36 trial staff (interviews); 110 (surveys)
If applicable, further details on the design or function of the proposed or actualized intervention	N/A
If applicable, details of the evaluative process for the proposed or actualized intervention	Main themes identified from interviews were used to investigate trial protocol suitability via quantitative survey
**Section 3: Outcomes**	
If applicable, outcomes measured (or anticipated outcomes)	Data suggests that protocol suitability can be increased by discussing and reviewing the protocol with trial staff in advance. Involving operationally experienced staff would be most useful. For multicentre trials, at least one trial staff member from each of the sites with the highest expected recruitment rates be involved in developing the protocol. Carefully assessing the context prior to study start is indispensable to ensuring protocol suitability and should particularly focus on the workforce and infrastructure available, as well as the needs and availability of trial participants. To allow for protocol suitability enhancing measures, planners must allocate enough time for trial preparation and solicit feedback and information on context at an early stage. Such prospective planning would increase implementability, efficiency and quality of trials in the long run.
Author’s conclusion as verbatim text:	
By applying an exploratory mixed methods approach, we identified a lack of clarity, implementability and adaptation to trial participants, workforce and infrastructure as the main constraints of protocol	

suitability. We found that site staff involvement in protocol development, careful assessment of local context, capacity and culture as well as ensuring that staff understands the protocol are the most helpful measures towards increasing protocol suitability, according to trial teams. Including technical aspects into such preparations and site involvement would simultaneously also enhance the feasibility of trials in the reviewed context. Considering and involving the site's input at an early stage of protocol development was deemed the best way to increase involvement, as the majority of trial staff did not seek major involvement in protocol development. Our data suggests that the measures presented increase implementability, efficiency and quality of trials in the long run, although it might slightly prolong the protocol development phase. We consider such an approach as particularly useful for clinical trials in SSA, as the protocols are mostly developed by Northern sponsors who might not be familiar with the setting.	
(Optional) References to other mentioned tools or studies of interest	N/A
**Section 4: Additional notes**	
Additional notes	Note: mixed-methods approach; assessed qualitative aspects on the JBI Qualitative Research Checklist.

**Section 1: Publication details**	
Author(s) or Organization(s)	Zuidgeest et al.
Title of publication	The GetReal Trial Tool: design, assess and discuss clinical drug trials in light of Real World Evidence generation
Link to publication	https://doi.org/10.1016/j.jclinepi.2021.12.019
Year of publication	2022
Geographic setting of the research (or, geographic affiliations of publishing authors)	Europe (Netherlands, Germany); UK
Funder and/or sponsor of the research	Innovative Medicines Initiative Joint Undertaking; European Union's Seventh Framework Programme
**Section 2: Intervention details**	
Type of intervention or proposed intervention to improve trial informativeness	GetReal Trial Tool (online interface)
Role of the intervention or proposed intervention within the trials research pathway	Allows users to assess the impact of design choices on generalizability to routine clinical practice, while taking into account risk of bias, precision, acceptability and operational feasibility.
If stated, domain of study per the authors (E.g., research waste, misconduct, feasibility...)	Evidence translation
Most relevant to Zarin et al? Or, other...	(2) Design: trial methods are likely to provide meaningful evidence related to the study hypothesis, and equally, (3) Feasibility: the trial is likely to be feasible
If applicable, population (or target population for the intervention)	Trialists (researchers)
If applicable, method(s) used to develop the intervention	The tool is grounded in the scientific literature combined with knowledge of experts from academia, pharmaceutical companies, HTA bodies, patient organizations, and regulators.
If applicable, sample size used to develop the intervention	Approx 30 people from 16 different organizations, including epidemiologists, statisticians, experts in clinical trial operations, and/or real-world evidence, patient representatives and ethicists, from academia, industry, patient organizations and SMEs.
If applicable, further details on the design or function of the proposed or actualized intervention	The tool combines information on trial design elements, their possible implications and possible operational challenges related to these (pragmatic) design choices.
If applicable, details of the evaluative process for the proposed or actualized intervention	The content of the tool is based on a combination of extensive literature review, in-depth stakeholder interviews, pragmatic trial study team conversations, and consortium member input. It has been validated by a team of trialists, clinical trial operational experts and epidemiologists. The tool functionalities have been piloted extensively, first before being made available online in June 2017 and again after revisions made to the tool in 2019.
**Section 3: Outcomes**	
If applicable, outcomes measured (or anticipated outcomes)	The current tool provides a navigation wheel with the seven domains, a visual aid on progress through the design choices, a two-level approach with the design choices and their possible implications on the first level and the operational challenges with their possible implications on the second level, an

	overview possibility per domain and a note-taking function. During trial design, the tool offers an easy to use interface, which supports users to navigate easily through different aspects of their trial design and reach a balanced decision on a design that is expected to be not only fit for purpose in theory but that also has the best chance of being successful in practice. The tool helps ensure all key aspects of the trial design and associated operational challenges are considered as a team builds and optimizes their trial design in light of a specific research question.<br><br>The tool will not make decisions for the user but rather highlight possible consequences of design choices, which need to be interpreted by the user to determine to what extent these consequences might apply to the specific trial the user is designing or evaluating, including the specific therapeutic area, intervention and health care setting. As such, the tool can best be used by a team with combined knowledge of clinical trial design, the disease area, health care setting and usual care options in scope for the specific research question.<br><br>The tool can also be of use at a later stage, when evaluating and communicating trial findings with key decision makers and the scientific community, by giving transparency into which design choices of the trial might have influenced generalizability and other aspects of the trial. The tool is also being regularly and successfully used in online educational courses on RWE and trial design.<br><br>It is explicitly not the aim of the tool to provide a "final score" regarding "level of pragmatism" for a trial, as the authors believe that each trial can only be evaluated taking into account the specific research question and context of the setting the trial aims to provide results for. In addition, similar color coding might have different implications for each trial, where for a sponsor with a limited budget higher costs might be a showstopper, while for another sponsor including more sites might be a more serious challenge.
Author's conclusion as verbatim text:	
In conclusion, the GetReal Trial Tool offers an accessible and solid knowledge base to assess, design or discuss clinical trials in light of RWE generation. The tool is open access and can be used without entering any confidential trial information. The tool can be found on www.getrealtrialtool.eu. The GetReal team welcomes any feedback on the functionalities and content of the tool, so that they can continue to optimize the tool.	
(Optional) References to other mentioned tools or studies of interest	N/A
**Section 4: Additional notes**	
Additional notes	N/A
