# Supplementary material for: Improving Trial Informativeness: A Rapid Review of Global Research on How to Ensure Trials Are Useful
Source: J Eval Clin Pract. 2025 Jun 11;31(4):e70147. doi: 10.1111/jep.70147 (PMC12158544; doi:10.1111/jep.70147)
Supplement: Supplementary file 3 — Supporting Material 3 JBI Checklists. [file JEP-31-0-s001.pdf]

JBI Checklist(s) (INFORM-RR)

JBI CRITICAL APPRAISAL CHECKLIST FOR TEXTUAL EVIDENCE: EXPERT OPINION https://jbi.global/sites/default/files/2023-09/2.Checklist_Textual_Evidence_Opinion.docx						
**Citation**	**Q1**	**Q2**	**Q3**	**Q4**	**Q5**	**Q6**
Alahdab & Murad (2018)	Y	Y	Y	Y	Y	N/A
Alphs & Bossie (2016)	Y	Y	Y	Y	Y	N/A
Bespalov et al. (2021)	Y	Y	Y	Y	Y	N/A
Brewster et al. (2021)	Y	Y	Y	Y	Y	Y
Calvert et al. (2018)	Y	Y	Y	Y	Y	Y
Chan et al. (2021)	Y	Y	Y	Y	Y	Y
Chang et al. (2023)	Y	Y	Y	Y	Y	Y
Clark et al. (2014)	Y	Y	Y	Y	Y	N/A
Dolley et al. (2024)	Y	Y	Y	Y	Y	Y
Heath et al. (2024)	Y	Y	Y	Y	Y	Y
Heath et al. (2021)	Y	Y	Y	Y	Y	Y
Jull et al. (2017)	Y	Y	Y	Y	Y	Y
Kavalci & Hartshorn (2023)	Y	Y	Y	Y	Y	Y
Khalil et al. (2022)	Y	Y	Y	Y	Y	Y
Lane et al. (2023)	Y	Y	Y	Y	Y	Y
Leiter et al. (2014)	Y	Y	Y	Y	Y	Y
Li et al. (2016)	Y	Y	Y	Y	Y	Y
Loudon et al. (2015)	Y	Y	Y	Y	Y	Y
McFadden et al. (2014)	Y	Y	Y	Y	Y	N/A
Meeker-O'Connell et al. (2016)	Y	Y	Y	Y	Y	Y
Natafgi et al. (2020)	Y	Y	Y	Y	Y	Y
Parks Taylor & Kowalkowski (2020)	Y	Y	Y	Y	Y	Y
Pirosca et al. (2020)	Y	Y	Y	Y	Y	Y
Robert et al. (2014)	Y	Y	Y	Y	Y	N/A
Sharma et al. (2017)	Y	Y	Y	Y	Y	Y
Suls et al. (2021)	Y	Y	Y	Y	Y	Y
Zuidgeest et al. (2022)	Y	Y	Y	Y	Y	Y

JBI CRITICAL APPRAISAL CHECKLIST FOR ANALYTICAL CROSS SECTIONAL STUDIES https://jbi.global/sites/default/files/2021-10/Checklist_for_Analytical_Cross_Sectional_Studies.docx								
**Citation**	**Q1**	**Q2**	**Q3**	**Q4**	**Q5**	**Q6**	**Q7**	**Q8**
Al-Durra et al. (2020)	Y	Y	N/A	Y	Y	Y	Y	Y
Bruckner et al. (2022)	Y	Y	N/A	Y	Y	Y	Y	Y
Taylor et al. (2023)	Y	Y	N/A	Y	Y	Y	Y	Y

JBI CRITICAL APPRAISAL CHECKLIST FOR QUALITATIVE RESEARCH https://jbi.global/sites/default/files/2021-10/Checklist_for_Qualitative_Research.docx										
**Citation**	**Q1**	**Q2**	**Q3**	**Q4**	**Q5**	**Q6**	**Q7**	**Q8**	**Q9**	**Q10**
Clayton et al. (2017)	Y	Y	Y	Y	Y	N	N	Y	N/A	Y
De Pretto-Lazarova et al. (2022)	Y	Y	Y	Y	Y	N	N	Y	Y	Y
Duley et al. (2018)	Y	Y	Y	Y	Y	N	N	Y	N/A	Y
Erber et al. (2021)	Y	Y	Y	Y	Y	N	N	Y	Y	Y
Jansen-van der Weide et al. (2018)	Y	Y	Y	Y	Y	N	N	Y	N/A	Y
Morgan et al. (2018)	Y	Y	Y	Y	Y	N	N	Y	U	Y
Swezey et al. (2020)	Y	Y	Y	Y	Y	N	N	Y	U	Y
Totton et al. (2023)	Y	Y	Y	Y	Y	N	N	Y	Y	Y
Toye et al. (2016)	Y	Y	Y	Y	Y	N	N	Y	Y	Y
Vischer et al. (2017)	Y	Y	Y	Y	Y	N	N	Y	Y	Y

Y – YES; N – NO; U- UNCLEAR; N/A – NOT APPLICABLE
